# Charge Carrier Processes and Optical Properties in TiO_2_ and TiO_2_-Based Heterojunction Photocatalysts: A Review

**DOI:** 10.3390/ma14071645

**Published:** 2021-03-27

**Authors:** Stefano Lettieri, Michele Pavone, Ambra Fioravanti, Luigi Santamaria Amato, Pasqualino Maddalena

**Affiliations:** 1 Institute of Applied Sciences and Intelligent Systems “E. Caianiello”, Consiglio Nazionale delle Ricerche (CNR-ISASI), Complesso Universitario di Monte S. Angelo, Via Cupa Cintia 21, 80126 Napoli, Italy; 2 Department of Chemical Sciences, University of Naples “Federico II”, Complesso Universitario di Monte S. Angelo, Via Cupa Cintia 21, 80126 Napoli, Italy; michele.pavone@unina.it; 3 Institute of Science and Technology for Sustainable Energy and Mobility, Consiglio Nazionale delle Ricerche (CNR-STEMS), Via Canal Bianco 28, 44124 Ferrara, Italy; ambra.fioravanti@stems.cnr.it; 4 Italian Space Agency (ASI), Space Geodesy Center “G. Colombo”, 75100 Matera, Italy; luigi.santamaria@asi.it; 5 Department of Physics “E. Pancini”, University of Naples “Federico II”, Complesso Universitario di Monte S. Angelo, Via Cupa Cintia 21, 80126 Napoli, Italy; pasqualino.maddalena@unina.it

**Keywords:** photocatalysis, charge lifetimes, absorption, photoluminescence, O_2_ sensing, composite photocatalyst, charge transfer, heterostructures, hydrogen production, ecological transition

## Abstract

Photocatalysis based technologies have a key role in addressing important challenges of the ecological transition, such as environment remediation and conversion of renewable energies. Photocatalysts can in fact be used in hydrogen (H_2_) production (e.g., via water splitting or photo-reforming of organic substrates), CO_2_ reduction, pollution mitigation and water or air remediation via oxidation (photodegradation) of pollutants. Titanium dioxide (TiO_2_) is a “benchmark” photocatalyst, thanks to many favorable characteristics. We here review the basic knowledge on the charge carrier processes that define the optical and photophysical properties of intrinsic TiO_2_. We describe the main characteristics and advantages of TiO_2_ as photocatalyst, followed by a summary of historical facts about its application. Next, the dynamics of photogenerated electrons and holes is reviewed, including energy levels and trapping states, charge separation and charge recombination. A section on optical absorption and optical properties follows, including a discussion on TiO_2_ photoluminescence and on the effect of molecular oxygen (O_2_) on radiative recombination. We next summarize the elementary photocatalytic processes in aqueous solution, including the photogeneration of reactive oxygen species (ROS) and the hydrogen evolution reaction. We pinpoint the TiO_2_ limitations and possible ways to overcome them by discussing some of the “hottest” research trends toward solar hydrogen production, which are classified in two categories: (1) approaches based on the use of engineered TiO_2_ without any cocatalysts. Discussed topics are highly-reduced “black TiO_2_”, grey and colored TiO_2_, surface-engineered anatase nanocrystals; (2) strategies based on heterojunction photocatalysts, where TiO_2_ is electronically coupled with a different material acting as cocatalyst or as sensitizer. Examples discussed include TiO_2_ composites or heterostructures with metals (e.g., Pt-TiO_2_, Au-TiO_2_), with other metal oxides (e.g., Cu_2_O, NiO, etc.), direct Z-scheme heterojunctions with g-C_3_N_4_ (graphitic carbon nitride) and dye-sensitized TiO_2_.

## Table of Contents

1. Introduction: why TiO_2_? ;                                  page 2
2. The role of TiO_2_ among photocatalysts.                            page 3
3. Historical facts.                                       page 7
4. Electronic properties and fundamental charge carrier processes in TiO_2_.              page 9
  4.1. Charge trapping and electronic states.                           page 9
  4.2. Electron trapping: trapping energies, nature of trap sites and time scales.           page 10
  4.3. Hole trapping: trapping energies, nature of trap sites and time scales.             page 13
5. Optical processes, charge recombination and photoluminescence in TiO_2_.             page 13
  5.1. Optical absorption.                                   page 13
  5.2. Electron-hole recombination and photoluminescence (PL) in TiO_2_.              page 17
    5.2.1. Relevance of photoluminescence analysis in TiO_2_.                   page 17
    5.2.2. Basic properties of charge carrier recombination and PL in TiO_2_.            page 18
    5.2.3. Anatase photoluminescence.                            page 19
    5.2.4. Rutile photoluminescence.                             page 22
  5.3. Interplay between photogenerated charges and molecular O_2_ adsorption: the O_2_-dependent PL. page 23
    5.3.1. O_2_-anatase interaction.                               page 23
    5.3.2. O_2_-rutile interaction.                                page 25
    5.3.3. Applications of O_2_-dependent PL of TiO_2_.                      page 26
6. Intrinsic TiO_2_ as photocatalyst: mechanisms and limits.                     page 28
  6.1. Basic photocatalytic processes and their characteristic times.                 page 28
  6.2. Limitations of intrinsic TiO_2_ as photocatalyst.                        page 30
7. Present and future trends for TiO_2_-based heterostructure photocatalysts and engineered TiO_2_.    page 31
  7.1. Engineered TiO_2_ nanocrystals.                              page 31
    7.1.1. Black TiO_2_.                                    page 31
    7.1.2. Facet engineered TiO_2_.                               page 34
  7.2. TiO_2_-based heterojunction photocatalysts.                         page 38
    7.2.1. TiO_2_/metal heterostructures.                            page 39
    7.2.2. TiO_2_/metal oxide semiconductors.                          page 41
    7.2.3. TiO_2_/g-C_3_N_4_ heterostructures.                            page 42
    7.2.4. Dye-sensitized TiO_2_.                                page 44
8. Conclusions.                                        page 46

## 1. Introduction: Why TiO_2_?

In the last three decades, titanium dioxide (TiO_2_, also named as titania) has been a major subject of study in materials science and technology for its functional properties and its versatility toward different applications. Hence, it can be reasonably said that TiO_2_ is—by itself—not a “novel” material. Nevertheless, the many interests in it have neither waned nor slowed down over the past few years. Why is this?

Several answers to such a question can be given, but ultimately most of them are related to its *photocatalytic properties* and to the potentialities of photocatalytic technologies as a tool to tackle some of the most important challenges in modern society, such as environmental and energy issues. Photocatalysis finds application in important fields such as—to name some of the most important examples—CO_2_ photoreduction [1,2], pollutant removal and pollution mitigation [3,4], hydrogen production via water splitting [5,6,7] or via photo-reforming of alcohols [8,9] and bactericidal activity [10,11,12]. Hence, *photocatalytic materials are widely regarded as keystones for addressing some major problems we will face in the near future*. Developing reliable, efficient, stable and unexpensive photocatalytic materials is thus regarded as a research field of great relevance.

TiO_2_ is undoubtedly one of the most widely studied and exploited photocatalysts, and often considered as a sort of benchmark in the field for the reasons that will be discussed next. Some of the contemporary “hot topics” in energy conversion and in light-based transformational materials/systems involve composites based on TiO_2_ (e.g., composites with g-C_3_N_4_, with graphene, with transition metal dichalcogenides, etc). In short, it can be stated that TiO_2_ represents an “evergreen” photocatalyst and—most importantly—that having a basic knowledge of the fundamental processes occurring in it is of paramount importance, especially for scientists working in the development of novel (TiO_2_—including) composite materials/systems.

A huge number of application-oriented papers is published every year on TiO_2_ and on TiO_2_-based composites. We think that it would be unrealistic (to say the least) to set up a review aiming at covering even briefly all the state-of-art related to the practical applications of TiO_2_-based materials. On another hand, the importance of papers that focus on some of the *fundamental processes* that define the electronic and/or photo-physical properties of TiO_2_ as an “intrinsic” metal-oxide semiconductor should not be underestimated, since these notions are often given for granted.

Based on these considerations, this review will be centered on *highlighting and reviewing some selected topics* for providing the reader with a *general picture of the basic electronic processes and optical properties* of TiO_2_. For example, photochemical and photoelectrochemical reactions on TiO_2_ surfaces are not discussed in detail in this work, considering that excellent reviews on these topics are already available in literature [3,13,14,15,16,17,18,19]. Instead, we will deal with fundamental notions and processes such as the position of excited and defect states, trap states, trapping/detrapping processes, optical absorption and radiative recombination (photoluminescence) and, in general, dynamics of photo-generated carriers and their interaction with environmental oxygen, as these are the fundamental processes that ultimately affect the applicative performances of pristine and modified forms of TiO_2_.

The present review is organized as follows:

In Section 2 and Section 3 we summarize the role of TiO_2_ among photocatalytic materials, highlighting the pros and cons of this material with also a brief historical introduction on the early findings of TiO_2_ photocatalytic properties.

In Section 4 we give an overview of elementary charge-carrier processes, i.e., electronic properties, in TiO_2_. The section focuses on single-particle properties of the charge carriers—namely energy levels, nature of the trapping states and their lifetimes. Separated discussions on electrons and holes are carried out for the sake of clarity.

Section 5 deals with optical properties and light emission (photoluminescence—shortened as PL) of TiO_2_, discussing why the study of such a phenomenon in controlled environment is interesting for photocatalytic materials and illustrating the dominant models and hypothesis that explain the fundamental mechanisms (i.e., radiative recombination channels) of PL in both anatase and rutile. The special case of the interplay between charge recombination and O_2_ adsorption is discussed, this being both a scientifically intriguing topic and a potentially useful phenomenon for O_2_ sensing.

Section 6 discusses the primary oxidation/reduction processes that define the photocatalytic behavior of TiO_2_ and the typical kinetics and lifetimes of the different steps of typical TiO_2_—based photocatalytic transformations in aqueous environment.

Finally, Section 7 summarizes the paper and briefly sketches some of the most recent routes currently employed to develop functional TiO_2_-based materials.

## 2. The Role of TiO_2_ among Photocatalysts

Based on the considerations made in the previous section, it is useful to start here by briefly summarizing what is meant by the term *photocatalyst* and which are the characteristics that a good photocatalyst shall exhibit.

A photocatalyst is any material that exhibits photocatalytic properties, i.e., the ability to foster and accelerate specific chemical reactions upon stimulation by light of suitable wavelength. The photocatalytic processes occur via the participation of free charges generated in the photocatalyst through quantum-mechanical transition of electrons into a mobile state via annihilation of the absorbed photons. As these free charges that diffuse toward the materials surface, they can then initiate redox reaction between reactants adsorbed at the photocatalyst surface. Hence, the “suitable” wavelengths mentioned earlier refers to wavelengths which are efficiently absorbed by the photocatalyst.

A material in which photogenerated free charge carriers (also “free carriers” for brevity) are present is not in a thermodynamically stable state: the photogenerated charges will remain in the excited (and mobile) state only for a transient amount of time (carrier lifetime) whose value ultimately depend on the probabilities associated to the various quantum-mechanical decay processes, including all possible non-radiative processes and spontaneous emission (i.e., radiative recombination or luminescence). Longer lifetimes imply greater probability that the free carrier will go into contact with the reagents and (possibly) foster a chemical reaction. From that it follows that conductors (metals) are not suitable as photocatalysts, due to their remarkably short lifetime associated to infra-band electron–electron scattering. Instead, photocatalysts of practical interest are typically semiconductors and, as a consequence, the light-induced free charge carriers involved in fostering redox reactions are electrons and holes belonging to the conduction and valence band (respectively) of the semiconductor.

**Figure 1:**
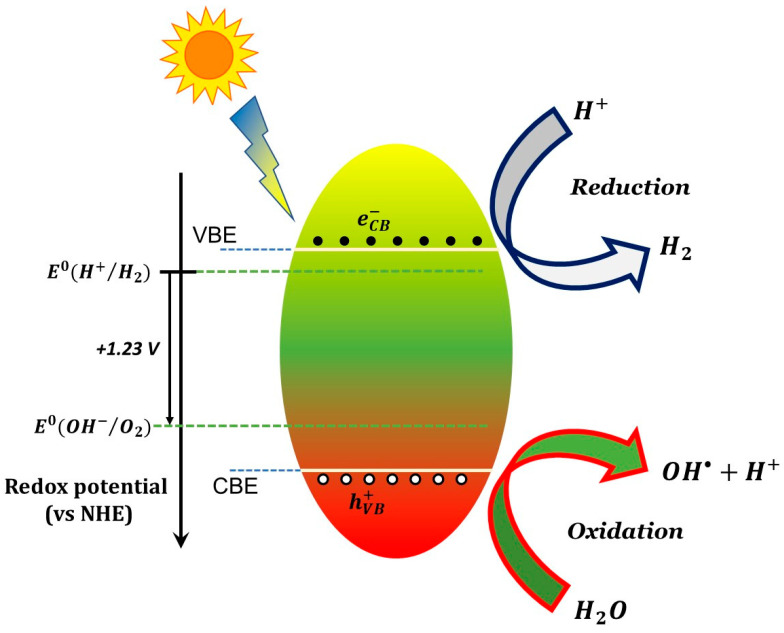
Elementary scheme for a basic photocatalytic process (here, water splitting) caused by illumination of a semiconductor photocatalyst. The scheme shows the reduction of H+ through electrons photogenerated in the conduction band and the oxidation of hydroxyl ions with formation of hydroxyl radicals through photogenerated holes in the valence band. In order for the two processes to occur the electrostatic potential associated to the conduction band edge (CBE) and valence band edge (VBE) have to be lower and greater (respectively) than the electrode potential of H^+^→H_2_ and OH^−^→O_2_ semi-reactions. The two half-reaction potential are separated by 1.23 Volts and thus a viable photocatalyst for water splitting shall have an energy gap of at least 1.23 eV. Larger gaps are actually needed to compensate for various sources of potential losses (“overpotential”). Similar considerations can be made for other redox reactions, using the corresponding electrode potentials.

**Figure 2:**
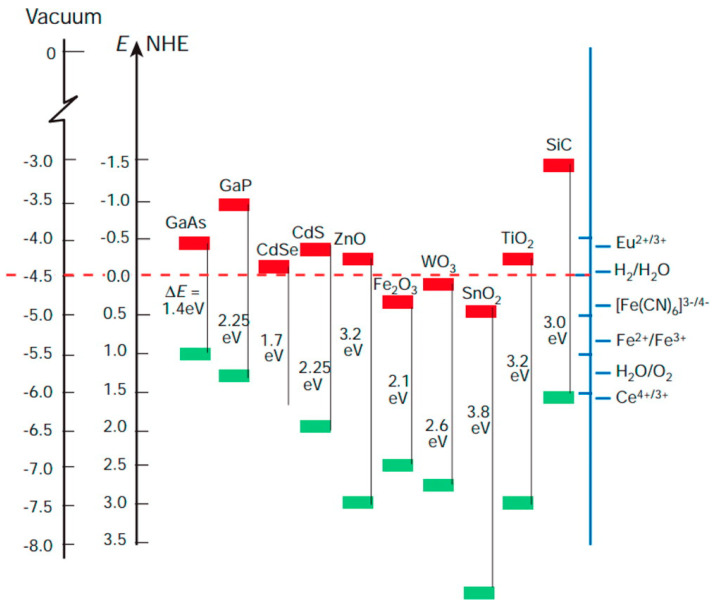
Energy position of conduction and valence band edges for selected semiconductors vs. various standard half-reaction potentials. The energy is represented in the vertical axis on the left) as referenced to the vacuum level and to the normal hydrogen electrode (NHE) level. Reprinted with permission from Reference [27]. Copyright 2014 American Chemical Society.

The photocatalytic action involves conduction band electrons as reactant-reducing agents and valance holes as reactant-oxidizing agents, as schematically shown in Figure 1 for a generic semiconductor. The efficiency of a specific redox reaction will depend on the relative values of the conduction (or valence) band energy of the semiconductor and the reduction (or oxidation) electrode potential of the specific half-reaction, as shown schematically in the caption of Figure 1. Additional examples of the energy position of conduction and valence band edges vs. various standard half-reaction potentials are reported in Figure 2.

A quantitative condition expressing the “suitability” of the wavelengths supposed to activate a given photocatalyst refers to the comparison between the quantum-mechanical energy *E* = *hν* of the photon and the semiconductor bandgap $E_{g}$. The photogeneration of free charges can occur only if $E>E_{g}$, i.e.,

(1) $$E=h\nu =\frac{hc}{\lambda }>E_{g}\Leftrightarrow \lambda \left(nm\right)<\frac{1240}{E_{g}\left(\mathrm{eV}\right)}$$

The simple relation in Equation (1) uses the numerical values of the speed of light (*c*) and of Planck constant (*h*) and indicates the optical “activation edge” of the photocatalyst. Radiation of wavelength longer than the band-edge value of Equation (1) are not suitable to activate photocatalytic effects, unless some specific modification of the material is made to induce additional sub-bandgap optical absorption (e.g., creation of defect states and/or sensitization with a different material). Obviously, activation edges lying in the far ultraviolet (e.g., UV-A and UV-B regions) are unpractical: photocatalysts working in this optical range would require artificial light, as UV-A and UV-B are blocked by the Earth atmosphere.

Although non-oxide semiconductors (e.g., transition metal-based monochalcogenides such as CdS and ZnS) and even completely different materials such as carbon quantum dots, MXenes and g-C_3_N_4_ also exhibit interesting photocatalytic properties, the most commonly employed photocatalysts are selected among metal oxides. Examples of photocatalytic metal-oxide semiconductors are represented by TiO_2_, ZnO, SnO_2_, CeO_2_, ZrO_2_, WO_3_, MoO_3_, Fe_2_O_3_ and Fe_2_O_3_.

There are plenty of available reviews on the photocatalytic properties of the above-mentioned materials, and here we will just mention some of the most recent ones, including some specifically relating with TiO_2_—based materials [20,21,22,23] and on other novel trends and topics of materials science for photocatalysis-related applications [24,25,26].

Apart from the mentioned issue of the optical activation energy, there are several other factors that can limit the photocatalytic efficiency of a semiconductor. The most important ones are:

(1) Large electron-hole recombination rates, as they would prevent the photogenerated charges from reaching the catalyst surface where the reaction between the adsorbed reactants shall occur.
(2) Susceptibility to photocorrosion, which decreases their photocatalytic efficiency.
(3) Scarce versatility of the material. By “versatility” here we mean the existence of reliable technological means to process/modify the material accordingly to the practical needs. For example: tuning of bandgap energy and/or modifying the optical spectrum of the material.
(4) Other practical issues, such as poor chemical stability, scarce biocompatibility, scarce affordability of the material synthesis/processing, etc.

A significant number of semiconductors have been investigated for their potential suitability as catalysts in photoelectrochemical reaction, including both oxides and non-oxides and with band gaps ranging from ≈1.4 eV (e.g., GaAs) to ≈3.5 eV (e.g., SnO_2_ [28]. Among them, TiO_2_ is widely studied and employed due to several reasons:

(1) It is cost-effective, i.e., can be produced at reasonable costs also in view of large-scale uses;
(2) It is photostable and resistant to corrosion;
(3) It shows a satisfactorily photocatalytic efficiency (at least once UV illumination is used for undoped TiO_2_, as will be discussed in more detail next);
(4) Several well-known chemical and physical techniques to produce TiO_2_ in form of porous films and nanoparticulate powders are available and well-developed [29,30,31,32,33,34,35,36,37];
(5) Finally, in recent years the problem of scarce activation by visible sunlight (that will be discussed next) has been mitigated: novel strategies of doping development of novel composites—some of which will be discussed in the present work—is allowing to obtain interesting results also in the field of visible light-activated photocatalysis.

Historically, TiO_2_ has been widely used as pigment for paints [38,39,40] and for other products such as toothpastes [41] and sunscreens [42,43,44]. A historical breakthrough took place with the discovery of photocatalytic water splitting on a TiO_2_ electrode illuminated by UV light [45], that brought large interest and efforts toward the scientific and technological research on titania. At the present days, the most important applications of TiO_2_ can be roughly classified in two categories, namely environmental and energy applications. For the reasons discussed in the next sections, these applications often rely on modified forms of TiO_2_, some examples including doped TiO_2_ and/or TiO_2_-based composites and heterostructures.

## 3. Historical Facts

As mentioned, the interest in TiO_2_ has been massively fostered by research in electrochemistry, and in particular by the mentioned 1972 achievement of photoelectrochemical water splitting by Fujishima and Honda [45]. It is to be noted that the position of the TiO_2_ band edges (vs. standard hydrogen electrode) indicates that conduction band electrons are good reducing agents toward hydrogen, whereas the valence band holes are strong oxidizing agents toward water (see Figure 2). This explains the light-induced decomposition of water into oxygen and hydrogen without the application of an external bias. Before that breakthrough, the photophysical and photo-transformative properties of TiO_2_ (and of other semiconductor metal oxides as well) had been already evidenced: here we briefly sketch an historical outline of this subject.

Titania has been used for centuries as pigment in paints. As a consequence, it has been recognized since long ago that it activates specific photochemical processes, manifesting in the “chalking” of exterior paints when exposed to intense sunlight [46,47]. By chalking it is meant here that a powder-like white substance tended to be formed and to fall away from the painting, in a fashion similar to the chalk on a blackboard. Even from this simple description we can recognize this phenomenon as a manifestation of what is today called “self-cleaning” property of TiO_2_. At the time, the chalking was correctly recognized as the effect of a partial removal of the organic component in the paint, leaving the TiO_2_ exposed. For example, an earlier report by Renz in 1921 discussed how TiO_2_ surfaces with adsorbed organic compound (such as glycerol) were partially reduced when exposed to sunlight. The reduction macroscopically manifested itself via a change in the color of titania, turning from white to grey, blue or black. Similar results were described in the same paper also for other metal oxides, such as Nb_2_O_5_, Ta_2_O_5_ and CeO_2_ [46].

Few year later, Baur and Perret reported about the photocatalytic deposition of a silver salt of ZnO to produce metallic silver [48], hypothesizing that the key element of the reaction was the photogeneration of free charges of opposite sign in ZnO that provided simultaneously oxidation of hydroxyl ions and reduction of Ag+ ions, i.e., $ZnO+\hbar \omega \to e^{-}+h^{+}$, $h^{+}+OH^{-}\to \left(1/4\right)O_{2}+\left(1/2\right)H_{2}O$ and $e^{-}+Ag^{+}\to Ag^{0}$.

In 1927, the ability of photoexcited ZnO to activate redox reaction was again invoked to explain the production of H_2_O_2_ from decomposition of formaldehyde [49]. Later studies on formation of metallic silver and gold confirmed that the these observed processes were indeed photocatalytic reductions activated by different light-exposed metal oxides, including Nb_2_O_5_ and TiO_2_ [50]. Other evidence of the specific photocatalytic characteristics of TiO_2_ were provided by study of photo-induced decompositions of dyes on TiO_2_ powder [51,52].

While during the 1950s the photocatalytic oxide of major interest was considered ZnO [53,54], the research on TiO_2_ was also developing. Kennedy and coworkers in a 1958 study on photo-adsorption of O_2_ underlined that photoexcited electrons were transferred to O_2_ then adsorbed as $O_{2}^{-}$ species on the TiO_2_ surface [55]. Such an ability to photo-adsorb the O_2_ was found to be correlated to the efficiency of photodecomposition of tested dye. Interestingly, some results also evidenced the possible involvement of oxygen lattice atoms of TiO_2_ in photocatalytic reaction of organic compounds. For example, a study of the photocatalytic oxidation of isopropanol to acetone on TiO_2_ suggested the following surface reaction [56]:

(2) $${\left(TiO_{2}\right)}^{*}+{\left(CH_{3}\right)}_{2}CHOH\to TiO+{\left(CH_{3}\right)}_{2}CO+H_{2}O$$

This process involves the formation of a surface oxygen vacancy, which can next be healed by dissociative adsorption of atmospheric O_2_, i.e., $TiO+\left(1/2\right)O_{2}\to TiO_{2}$.

The 1960s witnessed the publication of the first studies demonstrating the possibility to fully decompose (oxidize) organic compounds to water and CO_2_ via TiO_2_-based photocatalysis. One example was reported in 1965 by McLintock and Ritchie, who investigated the photo-decomposition of ethylene and propylene on TiO_2_ [57]. In the same decade, early photoelectrochemistry studies of TiO_2_ by Fujishima triggered interest as they evidenced that gaseous O_2_ could be evolved at electrode potentials referred vs. standard hydrogen electrode (“SHE”) quite shifted with respect to the expected values. For example, the experimental onset for O_2_ evolution obtained at about −0.25 V (vs. SHE) compared to the standard potential of +0.95 V in pH 4.7 aqueous buffer [58] indicated that the energy conversion (from photon energy to chemical energy) in TiO_2_ occurred with small losses, so that the photogenerated holes could react directly with water (according to the water photooxidation semi-reaction $2h^{+}+H_{2}O\to \left(1/2\right)O_{2}+2H^{+}).$

After the mentioned work of 1972 in which Fujishima and Honda reported about the ability to simultaneously generate hydrogen in a photoelectrochemical reactor (i.e., to achieve full water splitting), the field of photoelectrochemistry received a significant boost in attention and paved the way for the role of TiO_2_ in energy-based applications.

**Figure 3:**
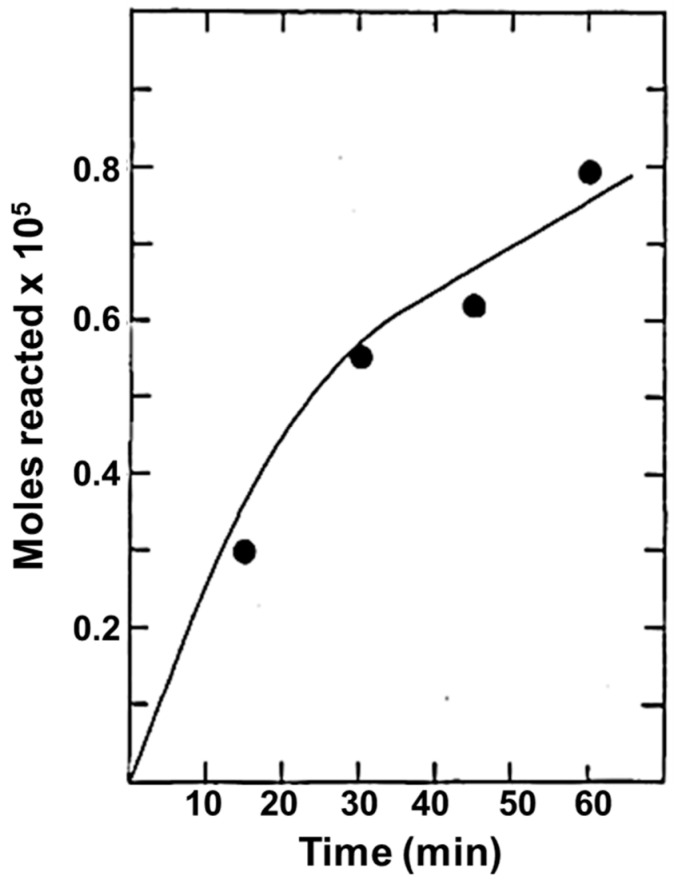
Number of moles of $CN^{-}$ that reacted after irradiation of an aqueous solution 1 mM $CN^{-}$ 0.1 M KOH with a xenon lamp while bubbling O_2_. Adapted with permission from Reference [59]. Copyright 1977, American Chemical Society.

We conclude this brief overview by also mentioning earlier reports on “environmental” applications. Works published 1977 by Frank and Bard studied the photocatalytic oxidation of cyanide and sulfite to cyanate and sulfate provided by photocatalytic activity of some semiconductor metal oxides, namely: TiO_2_, ZnO, Fe_2_O_3_ and WO_3_ [59,60]. An example of their experimental findings is reported in Figure 3, showing the data for the reaction of cyanide in aqueous solution due to photooxidation caused by anatase TiO_2_ [59]. The authors hence proposed the use of these photocatalyst for remediation of water via photo-induced decomposition of pollutants and focused in particular of photo-assisted oxidation of a number or organic and inorganic chemicals by polycrystalline TiO_2_ electrodes [61]. Since then, the non-selective oxidation of organic compounds/pollutants for water and air remediation is still an active field of study, as demonstrated by review papers published up to recent years. For example, we cite References [62,63,64,65] for water remediation and References [66,67,68] for air remediation.

The photodegradation of pollutants by TiO_2_ photocatalytic action typically requires the use of ultraviolet light. The practical experience demonstrated that the intensity of UV light present in the sunlight is typically non-sufficient to transform large quantities of organic compounds, and hence a medium/large scale application of the technology is somehow problematic. Such a limitation has oriented nowadays efforts and applications toward some specific directions. For example, attention has turned to cases in which a small UV intensity is enough to carry a limited amounts of reactions at the titania surface such as, for example, the anti-bacterial disinfection of TiO_2_—treated surfaces [69]. An obvious consequence of this limitation is that much research efforts are focused on developing TiO_2_—based photocatalysts that can be activated by visible light, as will be discussed next.

## 4. Electronic Properties and Fundamental Charge Carrier Processes in TiO_2_

Titania is in most cases found (or synthesized) in either anatase or rutile phase. Anatase is a meta-stable phase which irreversibly convert to rutile at temperatures larger than 600 °C, while the rutile phase is thermodynamically stable. Anatase is the most active photocatalytic phase, a fact typically attributed to larger concentrations of surface hydroxyl and hence to an improved (compared to rutile) generation of $HO^{•}$ and $H_{2}O_{2}$ species [70]. It is worth mentioning that another meta-stable phase exists, namely brookite whose photocatalytic properties have not been investigated in much detail [71,72,73]. In the rest of Section 4 we will sometimes use the abbreviations A-TiO_2_ (or just “A”) and R-TiO_2_ (or just “R”) to indicate the TiO_2_ anatase and rutile phase (respectively). Some of the bulk properties of the two polymorphs are reported in Table 1, which reports data taken from the Oxide Handbook by Samsonov [74], unless noted otherwise.

### 4.1. Charge Trapping and Electronic States

The absorption of photons whose energy is at least equal to the bandgap of the semiconductor induces to the creation of a pair of “free” (i.e., mobile) charges in the valence (a hole) and in the conduction band (an electron) of the semiconductor. Next, the fate of the free charges (and, in particular, whether they can reach the semiconductor surface or not) depends on the probability of recombination and of charge trapping processes. Both of these processes typically depend on the morphology and on the defect configuration (i.e., the type and the concentration of the defects) of the semiconductor nanoparticle.

Charge trapping indicates a generic process of electronic relaxation toward a localized state, typically belonging to the bandgap region of the semiconductor. Once trapped, the charge is of course no longer mobile. The trapped electrons (or holes) whose energy distance to the edge of the conduction (of valence) band is close to k_B_T (where k_B_ is the Boltzmann constant and T is the temperature) can also be detrapped, that is they can return to a mobile state and continue to move in the crystal. The trapping process is however not reversible if it occurs into a deep trap, i.e., when charges are trapped in sites associated to electron states whose energy distance from the band edges is much larger than k_B_T. In such a case, the most likely fate of these charges is to recombine non-radiatively. For this reason, deep traps are often referred to as recombination centers.

The charge mobility of a semiconductor is clearly a crucial parameter in order to assess its efficiency as photocatalyst. The reactants are in fact typically present as adsorbed species on the semiconductor surface, and redox processes can occur only when started (triggered) by a charge that reached the surface. Based on what we said about the trap states, it can be broadly said that the presence of deep charge traps is detrimental to its photocatalytic efficiency. However, to assume this as broadly valid statement would be simplistic. For example, trapping could be also considered beneficial when it localizes charge carriers at sites which are preferential for the adsorption of potential reactants, or if promotes charge separation.

### 4.2. Electron Trapping: Trapping Energies, Nature of Trap Sites and Time Scales

Electron trapping is quite important for TiO_2_ photophysics. In fact, most of the photogenerated electrons are expected to be already trapped long before they initiate a reduction reaction. As discussed in Section 6, in fact, the timescale for electron trapping is of the order of few nanoseconds.

Studies involving direct and/or indirect measurements or the binding energy of shallow and deep electron traps in TiO_2_ report values in an energy range between 0.1 and 0.8 eV [76,77,78,79,80,81,82,83]. consistent with photoemission results for electronic defects on the surface of TiO_2_ single crystal surfaces [15].

**Figure 4:**
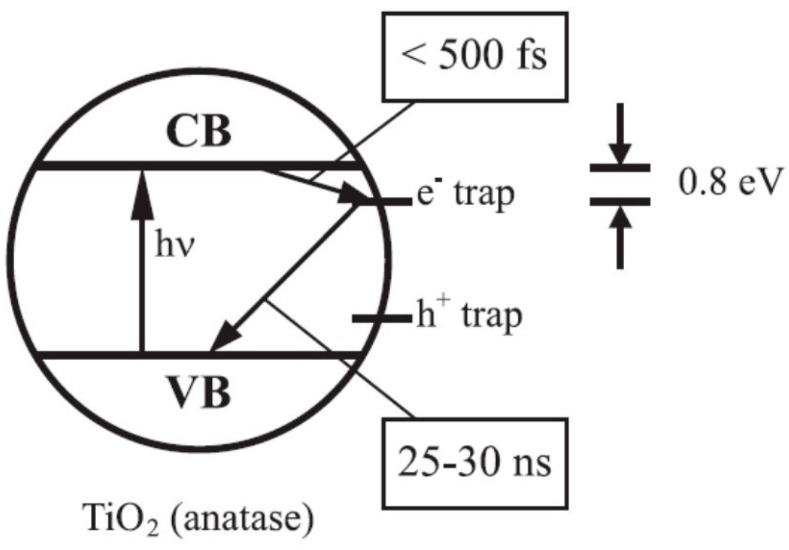
The scheme proposed for the dynamics of photogenerated electrons by Leytner and Hupp, according to the results of their time-resolved photoacoustic spectroscopy experiments. The free electrons are trapped via a fast process in states which are found to have an average energy 0.8 eV below the conduction band edge, so that they shall be considered deep traps. While the position of traps is in good accord with photoluminescence experiments, the lifetime of such trapping process was estimated to be less than 1 ns, in partial disagreement with the scheme proposed by Hoffman and coauthors [84] (discussed in Section 6.1) which associated processes of such rapidity to shallow traps. Reprinted from Reference [76] with permission from Elsevier. Copyright 2000 Elsevier Science B.V.

For example, Leytner and coworkers [76] employed a time-resolved version of photoacoustic spectroscopy to study colloidal TiO_2_ nanocrystals in aqueous solutions, finding evidence of electron traps states, available to photoexcited conduction electrons, whose energy levels lie below approximately 0.8 eV below the conduction band edge, as shown in Figure 4. In this work the decay of trapped electrons to the valence band was estimated to occur with a lifetime of about 25–30 ns and an average photon energy of 3.2−0.8 = 2.4 eV, in good agreement with the experimental finding for the “green” photoluminescence of anatase, discussed in Section 5.2.3.

It is worth noting that other works evidence the presence of surface states at the contact between TiO_2_ (rutile, generally) electrodes and electrolyte solution. Earlier analyses conducted by Siripala and Tomkiewicz [77] on the photocurrent induced by sub-bandgap illumination of single-crystal TiO_2_ electrodes in milder acidic conditions (pH = 6.5) evidenced electron promotion from the valence band to gap states lying around 0.6 eV below the bottom of the conduction band. Since then, several works evidenced comparable results regarding the energy of the infra-gap traps and surface states, For example, surface states positioned at an energy 0.7 eV below the conduction band edge in TiO_2_ in nanocrystalline TiO_2_ electrode in acidic conditions (pH = 3) were reported by Redmond and coworkers [78], while Boschloo and coauthors evidenced surface states of nanostructured anatase TiO_2_ via spectroelectrochemical methods located about 0.5 eV below the conduction band edge [79,80].

**Figure 5:**
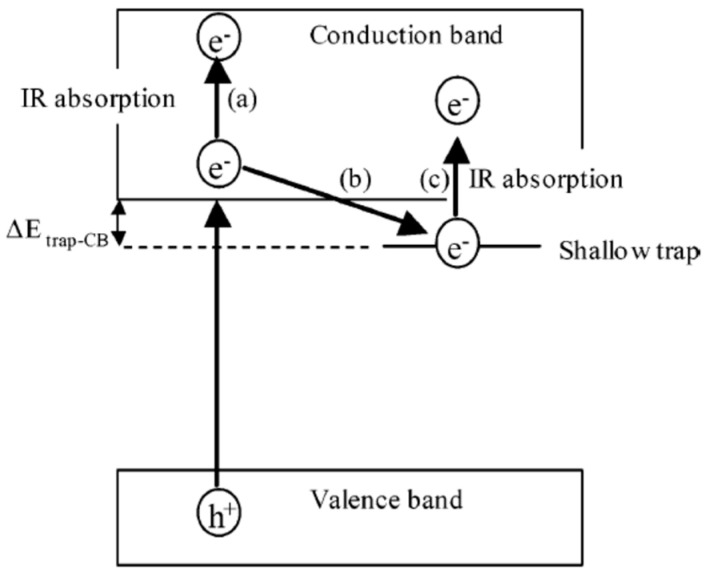
Processes identified by infrared spectroscopy in Reference [82] on Degussa P25 TiO_2_ after the removal of adsorbed water. Spectroscopic analysis of the excitation of electrons trapped in shallow traps (process (c) in figure) provides an energy distance of about 0.1 eV between the conduction band and the trap states. Reprinted with permission from Reference [82]. Copyright 2004 American Chemical Society.

Other works highlighted the presence of shallow traps also. Experiments by Warren and coworkers [82] conducted in controlled atmosphere on dry TiO_2_ indicated that the de-hydrated powders produced a broad infrared spectrum peaked at 880 cm^−1^, attributed to shallow traps present at 0.1 eV below the conduction band (see Figure 5).

To summarize, traps states are typically found within an interval of energies, in most cases reported to be centered at about 0.5–0.7 eV for the deep traps and 0.1 eV for the shallow traps. As origin of the dispersion of the allowed trap energies the heterogeneity of trap sites is often invoked [85,86,87,88]. Many researchers also agree that electrons are preferentially trapped at the surfaces for both anatase and rutile TiO_2_ [81,83,87,88,89,90,91,92,93,94].

Although less often mentioned, surface OH groups have also been proposed as alternative trapping sites of photoexcited electrons. For example, this was proposed by Szczepankiewicz and coauthors [95], based on Fourier transform infrared (FTIR) experiments. The same possibility has also been mentioned by Henderson and coworkers for single-crystal rutile [96,97]. However, also bulk (i.e., internal) sites [98,99,100,101,102] and grain boundaries of sintered films [103] have been reported as possible trap sites for electrons.

As the oxidation state of Ti in a stoichiometric TiO_2_ crystal is 4+ (indicated as Ti^4+^ or Ti(IV)), a Ti^3+^ site is synonymous of an electronic excess, which might arise from the trapping of a photoexcited electron (i.e., $e_{CB}^{-}+Ti^{4+}\to Ti^{3+}$) or to the coordination of the Ti spaces with an oxygen vacancy (V_O_), which are typically associated to localized electronic charge. While it is generally true that a neutral OV accommodates electrons (two in the case of a V_O_ in TiO_2_), the question of their actual degree of localization (in other words, the spatial extent of their orbital) depends on the actual strength of the ionic bonding and interactions vs the possibility of the crystal to lower its energy by relaxing in a much different bonding configuration [104].

It might be tempting to assume that the degree of localization of an electron trapped at a Ti^3+^ site trapped charge could be estimated by adsorbing on the surface an electron-scavenging species (e.g., O_2_) and observing whether their reaction occurs or not as a function of the distance between the surface and the trap center. However, the degree of electronic delocalization around the trap site could be sufficient to allow an electronic coupling with the surface O_2_ species even when the trap is not at the surface. For example, Wendt and coworkers performed this kind of analysis for rutile TiO_2_ via scanning tunnel microscopy (STM) measurements, arguing that even sub-surface charge associated with Ti^3+^ interstitial sites can interact with ambient O_2_ molecules and promoting their dissociative adsorption [105]. In conclusion, it is probably fair to state that the depth at which electrons trapped at Ti^3+^ sites can no longer participate in surface chemistry is not known. As a consequence, there is no easy way to assess whether an electron trap close to the surface but not at the surface (i.e., a sub-surface trap) is beneficial or not for the photocatalytic activity.

As mentioned previously, the photo-reduction processes such as the formation of superoxide radicals (Equation (7)) are expected to be mostly caused by trapped electrons. In fact, many studies concur that the trapping of a photogenerated occurs on a sub-picosecond time scale and possibly shorter than 100 fs [98,106], while instead its detrapping typically occurs on a much longer timescale.

More in detail, experimental studies indicate that the lifetimes of a trapped electron in TiO_2_ have a large variability [81,95,107,108,109,110,111] depending on the eventual presence (or absence) of electron scavengers (e.g., O_2_) and/or hole scavengers.

**Figure 6:**
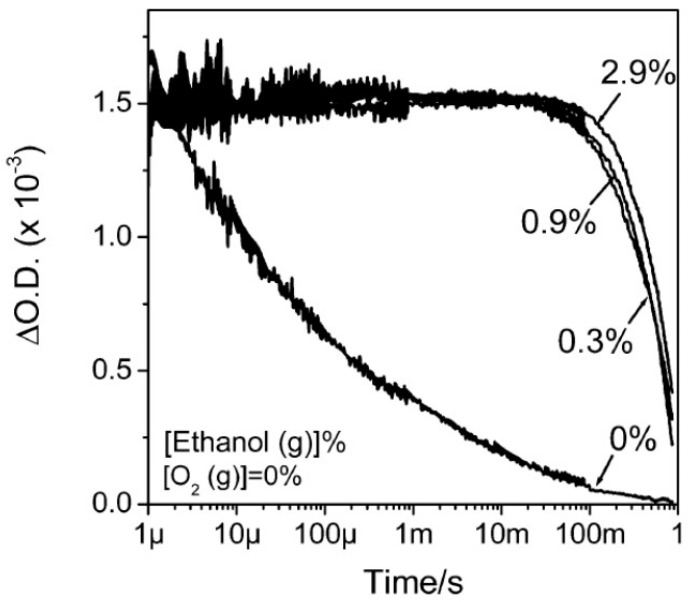
Time-resolved transient absorption signal at probe wavelength 800 nm for TiO_2_ nanocrystals in anaerobic conditions. The percentages indicate the concentrations of ethanol (ranging from 0 to 2.9%). Pump (excitation) wavelength at 337 nm. Reprinted from Reference [112]. Copyright 2006 American Chemical Society.

Trapped electron lifetimes spanning over timescales from hundreds of picoseconds [81] to tenths of microseconds [112] in “non-perturbed” TiO_2_, while they can be noticeably much longer if an hole scavenger (such as ethanol) removes the possible recombination partners of the trapped electrons. An example is reported in Figure 6 (reprinted from Ref. [112]) whose authors performed transient absorption spectroscopy on commercial P25 and P90 nanocrystalline films (the latter has greater anatase content and smaller particles than the former). The optical absorption at 800 nm (~1.55 eV) was used as a probe of the excitation of trapped electrons, while ethanol was used as hole scavenger. As shown in Figure, in absence of both ethanol and oxygen a lifetime of about 25 μs, while in presence of ethanol the lifetime increased of a factor of six order of magnitudes (~0.5 s).

Moreover, it has also been reported that under ultra-high vacuum conditions (that is, the best controlled conditions to exclude the intervention of any charge scavenger), localized electronic charge associated to Ti^3+^ sites on single crystal TiO_2_ surfaces can persist indefinitely [15].

The ability to durably store trapped electrons at a given concentration is an important characteristic for defining the photocatalytic efficiency of a catalyst. In the case of nanoparticulated TiO_2_, an early study indicated that up to about 10% of the Ti^4+^ sites could be occupied by a trapped electron if EtOH was used as hole scavenger to reduce the probability of electron-hole recombination [113]. The results of this study were later strengthened by other findings indicating trapping capacity in the range of 7–14% [109,114,115]. In terms of electron densities of surface traps, values have been found in literature as ranging in a typical interval of 10^12^ to 10^13^ cm^−2^. For example, Wang and coworkers [116] reported surface concentrations of trapped electrons varying as a function of the pH from about 5 × 10^11^ cm^−2^ at pH = 4.7 to about 5 × 10^13^ cm^−2^ at pH = 13. These values are consistent with that of ~ 3 × 10^12^ cm^−2^ reported by Boschloo [80]. It is not completely clear whether the density of trapped electrons is correlated with the specific surface area. For example, Katoh and coworkers [117] showed that TiO_2_ particles of 20 nm diameter trapped more electrons per unit surface than particles of 300 nm diameter. However, this conclusion is challenged by other experimental findings [81].

### 4.3. Hole Trapping: Trapping Energies, Nature of Trap Sites and Time Scales

Theoretical predictions based on electrostatic calculations [118] on hole and electron trapping at the TiO_2_(110) surface of rutile indicated near-surface sites as the preferred to bulk sites for hole trapping in rutile TiO_2_, a conclusion that is consistent with results by Shapovalov et al. [119] using an ab initio embedded cluster approach. Experimental investigations on the sites of hole trapping have often employed electron paramagnetic resonance (EPR). A consensus exists on the fact that the most likely trap sites are surface undercoordinated surface atoms (i.e., Ti^4+^-O surface terminations) [120,121,122,123].

Concerning the time dynamics of hole trapping, investigations indicate that on TiO_2_ surface it occurs at about the same rate as electron trapping. For example, one analysis [124] conducted via transient absorption spectroscopy in anatase particles estimated a hole trapping time of about 50 fs, while other works report hole trapping timescale of ∼200 fs for ‘hot’ holes resulting from band-to-band excitation [125,126], followed by thermalization over about successive 0.1 ns.

Other works worth mentioning are those from Furube’s group in which transient absorption spectroscopy (in combination with the use of methanol as a scavenger) was employed to track the hole trapping in nanocrystalline films of A-TiO_2_ [126,127]. These authors assigned the spectral region at ~500 nm to excitation events associated with trapped holes, similarly as Yang and Tamai [124] who observed a broad transient absorption signal at ∼520 nm evolving immediately on excitation of colloidal A with a 200 fs 360 nm pulsed laser source. This absorption was assigned to excitation of trapped holes because it was not observed if the hole scavenger SCN−was present. Interestingly, this absorption is very close in photon energy to the transitions correlated to the “green photoluminescence”, discussed later in Section 5.2.3, which are also attributed to trapped holes.

## 5. Optical Processes, Charge Recombination and Photoluminescence in TiO_2_

### 5.1. Optical Absorption

**Figure 7:**
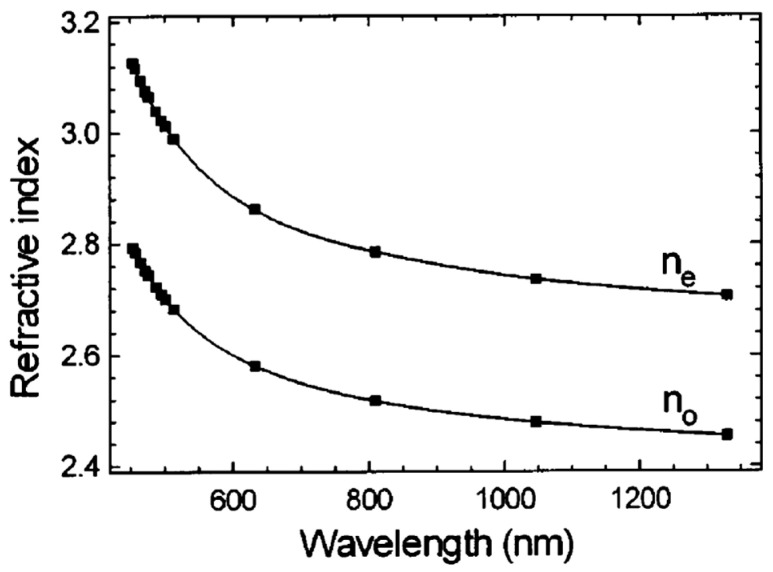
Experimental values of the ordinary (n_o_) and extraordinary (n_e_) refractive index of rutile TiO_2_ crystals as a function of wavelength at temperature T = 30 °C. The continuous curves are calculated via a Sellmeier-like interpolation. Reprinted with permission from Reference [128]. Copyright 1997 American Institute of Physics.

A-TiO_2_ and R-TiO_2_ are both transparent in the visible and near-infrared range, with a transmission interval of about Δλ = 0.4–7 μm and birefringent, as they belong to the tetragonal system. The rutile has positive birefringence, meaning that its extraordinary refractive index is larger than its ordinary index. It exhibits an index difference $\Delta n=n_{e}-n_{o}$ of about 0.27–0.29 in the visible range (see Figure 7). On the other hand, anatase shows a negative birefringence.

**Figure 8:**
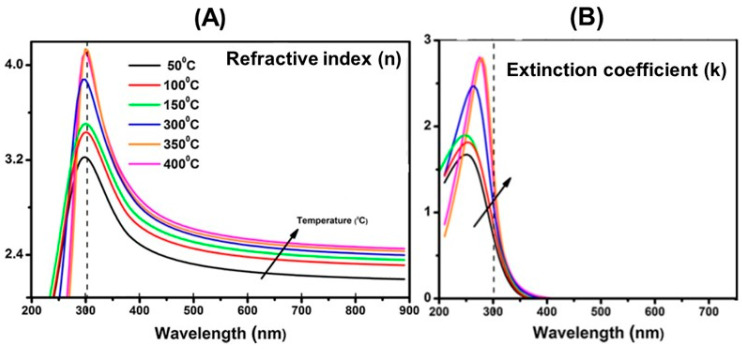
Plots of refractive index (**A**) and extinction coefficient (**B**) of amorphous TiO_2_ films (grown at 50, 100 and 150 °C) and of nanocrystalline TiO_2_ films (grown at 300, 350 and 400 °C) films. The black arrow indicates the increase of n and k with increasing growth temperature. In all cases it is clearly evidenced the absence of optical absorption for wavelengths larger than 400 nm. Reprinted from Reference [131] with permission from Elsevier. Copyright 2014 Elsevier Science B.V.

The refractive properties of R-TiO_2_ have been investigated in some detail, as it serves in practical applications. Specifically, R-TiO_2_ is commercially available as large crystals of good optical quality and large refractive indexes. As shown by the m-lines measurements by Rams and coworkers [128], the extraordinary refractive index $n_{E}$ of R-TiO_2_ in the wavelength interval Δλ=1350−450 nm varies from (about) $n_{E}\left(1350\;\mathrm{nm}\right)\approx 2.70$ to $n_{E}\left(450\;\mathrm{nm}\right)\approx 3.13$, while for the ordinary index $n_{O}$ we get $n_{O}\left(1350\;\mathrm{nm}\right)\approx 2.45$ and $n_{O}\left(450\;\mathrm{nm}\right)\approx 2.80$ (Figure 8).

Due to such a large refractrive index, rutile prisms are often used in optical laboratories to couple the light into optical waveguides. This requires a precise knowledge of its refractive and thermo-optical coefficients. An earlier work by Devore (Ref. [129]) is also quite useful in estimate the dispersive refractive indexes of R-TiO_2_, as they provide a good extrapolation using a Sellmaier equation of the form:

(3) $$n_{k}^{2}\left(\lambda \right)=A_{k}+\frac{B_{k}}{\lambda^{2}-C_{k}}$$

where the suffix “k” indicates either ordinary or extraordinary wave. The best-fit values for the coefficients A, B and C are reported to be $A_{e}=7.197,\;B_{e}=0.3322\;\mathrm{and}\;C_{e}=0.0843$ for the extraordinary wave and $A_{o}=5.913,\;B_{O}=0.2441\;\mathrm{and}\;C_{e}=0.0803$ for the ordinary wave [129].

Measurements of dispersive refractive index of anatase are less available [130], probably due to the difficulty of preparing good quality anatase crystals and to the fact that anatase is actually much more useful and employed in form of nanoparticle films and powders due to its superior (respect to R-TiO_2_ ) photocatalytic properties.

Figure 8 reports experimental values of the refractive index (n) and extinction coefficient (k) in the UV-visible range obtained via spectroscopic ellipsometry for amorphous and nanocrystalline thin TiO_2_ films grown at different substrate temperatures ($T_{S}$) on Si(100) substrates via Atomic Layer Deposition [131]. The data indicated refractive index values increasing from amorphous ($T_{S}\leq 150$ °C) to nanocrystalline films 250 °C $<T_{S}\leq 400$ °C. All measurements evidence in a clear way the negligible occurrence of optical absorption in the visible range.

The bulk optical absorption properties of A and R have been studied in the past in great detail [130,132,133,134,135,136,137,138]. Their importance is underlined by the fact that they represent the first step (i.e., the photogeneration of free charges) of the photocatalytic process. The optical absorption spectra of the two main polymorphs of TiO_2_ have been obtained by different techniques, including photoacoustic spectroscopy [76] and photoconductivity [134,139].

**Figure 9:**
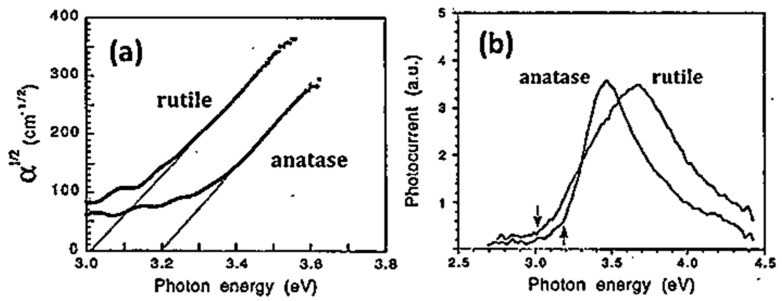
(**a**) Optical absorption spectra of anatase and rutile films measured at room temperature and plotted as α^1/2^ (cm^−1^ unites) vs. photon energy (eV units). The graph evidences the different indirect bandgap energy of the two polymorphs. (**b**) Photoconductivity spectra of anatase and rutile films. The arrows indicate the optical absorption edges. The anatase sample evidences a broader energy distribution of shallow donors in the sub-bandgap region. Adapted from Reference [134] with permission from Elsevier. Copyright 1994 Elsevier Science B.V.

As also indicated in Table 1, the optical absorption edges (i.e., bandgap energies) of A and R occur at room temperature at photon energies of about 3.2 eV and 3.0 eV, respectively [140]. The photon energy values of optical absorption edges increase as the temperature of the crystals is decreased for both polymorphs [135]. It is generally held that they bandgaps in bulk R and A are direct and indirect, respectively. The threshold for the first direct bandgap transition in A is reported to be at ∼3.8–4.0 eV [141,142]. In this same range (i.e., ~4.0 eV) the maxima of the bulk optical absorption spectra of both polymorphs also occur [143,144]. A detailed study of the optical absorption behavior of anatase TiO_2_ at photon energy lower than absorption edge has been conducted by Tang and coworkers [134,136], who showed (see Figure 9) that the absorption coefficient exhibits a Urbach tail, i.e., a spectral region in which it increases exponentially vs. increasing photon energy [145] at all temperatures.

The same authors studied the dichromic behavior (i.e., the difference in the absorption properties perpendicular to versus parallel to the c-axis) of the two phases, showing that it increases with temperature in anatase, while disappears in R while approaching room T [136]. A possible interpretation of this effect was provided by a first-principles calculation indicating that non-bonding dxy orbitals preferentially oriented perpendicular to the c-axis are present at the bottom of the anatase CB, contributing to its anisotropic optical absorption [146].

Observation of quantum-size effects (quantum confinement) in the absorption spectra of ultra-small TiO_2_ nanoparticles is controversial. Works have been published reporting both the occurrence [147] and the absence of quantum confinement effect [148,149]. For example, a blueshifts in the absorption threshold of anatase TiO_2_ as a function of decreasing particles sizes below 2 nm was reported by Satoh and coworkers [147], who stated that the experimental results matched their calculations based on the effective mass approximation. On another hand, no bandgap shift for anatase nanoparticles of diameters down to 2 nm was evidenced by Serpone and coworkers [148], who suggested that absorption edge blueshifts observed for anatase nanoparticles below 2 nm diameter shall be assigned to a transition from an indirect to direct bandgap excitation, an effect caused by geometric distortions of Ti coordination occurring when anatase particles became ultra-small.

**Figure 10:**
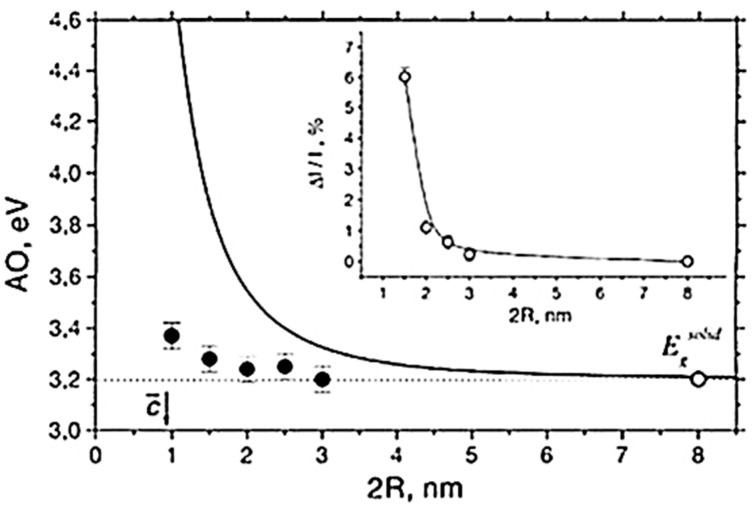
Absorption onset energy of colloidal TiO_2_ nanoparticles of different diameters (3, 2.5, 1.5 and 1 nm). The solid line is a theoretical calibration curve based on effective mass approximation. The inset reports relative changes of the (101) lattice constant calculated by XRD spectra. Reprinted from Reference [149] with permission from Elsevier. Copyright 2000 Elsevier Science B.V.

Monticone and coworkers [149] also examined the optical properties of small anatase nanoparticles of different particle size distribution, observing little or no blueshift for particles with diameters from 3 nm down to 1 nm (see Figure 10). The data did not fit the effective mass approximation and did not support the occurrence of quantum size effect in TiO_2_ nanoparticles. Moreover, the same authors observed changes in the oscillator strengths of optical transitions in the 3.5–4.5 eV range with decreasing particle, which they did not attribute to quantum size effects but to increased lattice strain in the particles.

**Figure 11:**
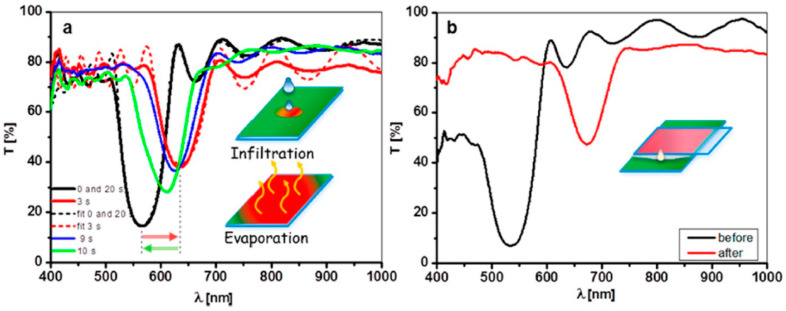
Example of the spectral reflectivity and chemical sensing capabilities of a 1D photonic crystal (multilayered reflector) obtained by stacking thin layer of controlled thickness and porosity by pulsed laser deposition. (**a**) Measurements of transmission spectra of the TiO_2_ photonic crystal before and after infiltration with acetone. The transmission measurements are modified due to the modification of the refractive index caused by infiltration of acetone the pores, followed by its evaporation. (**b**) Modification of the transmission spectrum caused by infiltration with a liquid crystal. Reprinted with permission from Reference [151]. Copyright 2014 American Chemical Society.

**Figure 12:**
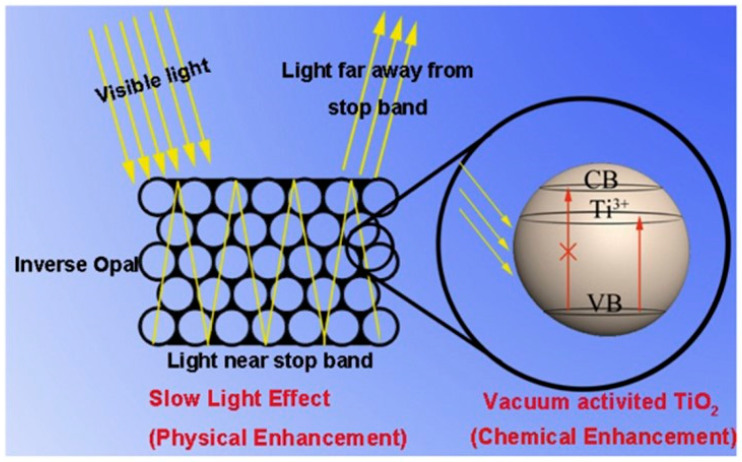
Illustration of the synergistic effect of the photonic resonances in an inverted-opal TiO_2_ photonic crystal and its photocatalytic activity. The off-resonance light is mostly reflected away, while the light belonging to the photonic stop-band is slowed down (“slow light effect”) and optical modes with larger intensity on the photocatalyst (the high-index regions) are formed leading to an efficient excitation of the photocatalyst. Reprinted from Reference [156] with permission from Elsevier. Copyright 2014 Elsevier B.V.

It is known that multilayered structures that alternate low and high refractive index layers can be engineered to form efficient reflectors [150], whose optical properties are mainly dictated by the periodicity of the optical thickness of the layers and on the refractive index differences between low and high index layers. Thanks to its large refractive index and to the variety of techniques allowing to process and micro-engineering TiO_2_ structures, multilayered reflectors [151] (Figure 11) and, more in general, TiO_2_-based three-dimensional photonic crystals have been fabricated and investigated, proving to be useful in different ways. For example, photonic resonances can be used to localize the electromagnetic energy at specific frequencies (“slow light” effect) in the high-refractive index photocatalyst and enhance the photoinduced, as schematically shown in Figure 12. Activation enhanced hydrogen production in photoelectrochemical cells was reported by Chiarello and coworkers [152] using a TiO_2_ photonic crystal as photoanode.

Applications of TiO_2_ in chemical sensing, SERS detection and photocatalysis have been reviewed in Refs. [23,153,154,155].

### 5.2. Electron-Hole Recombination and Photoluminescence (PL) in TiO_2_

#### 5.2.1. Relevance of Photoluminescence Analysis in TiO_2_

In the context of studies of a catalytic material it is of paramount importance to gather information on the active surface sites of the catalyst and on how they affect the dynamics of adsorption and photoactivated transformations of the targeted species. In this regard, studies of PL properties of the catalyst in controlled environment and during a reaction are very well suited and useful.

PL phenomena in semiconductors are driven by diffusion and recombination of photogenerated charges, that typically occurs in a thin region beneath the semiconductor surface (typical widths of few tenths of nm if the excitation is provided at supra-gap photon energy), making it very sensitive to small local variations. Even if PL is not intrinsically surface-selective as special techniques such as surface second harmonic generation [157,158,159,160], it has the advantage that it is usually not difficult to obtain a fairly good signal-to-noise ratio, furthermore in absence of background signal that are present in other optical spectroscopy techniques (e.g., reflectivity).

Moreover, PL techniques involve lifetime measurements and allows dynamic analysis during the reactions (i.e., while the catalyst is in contact with the reactant and as the pressure of the reactant is changed in a controlled manner). Finally, as already mentioned in the previous section, the PL efficiency gives indications on the lifetimes of charge carriers and therefore it usually correlates with the photocatalytic efficiency.

As mentioned, M. Anpo and coworkers were among the first who systematically employed photoluminescence as one of the standard techniques of study of photocatalytic materials and of the redox transformations occurring on their surfaces [161,162,163,164,165]. Since then, PL has been often employed as an indirect test to assess the usefulness of modifications of a material (for example, of heterogeneous doping), as a dramatic quenching of the PL signal would typically indicate an improvement in the photocatalytic properties.

#### 5.2.2. Basic Properties of Charge Carrier Recombination and PL in TiO_2_

Recombination represents the conclusive process for photogenerated electrons and holes. The excess (with respect to equilibrium) energy associated to the excited free charge carriers can be dissipated via emission of a photon (radiative recombination) or by other mechanisms (non-radiative recombination). Radiative recombination events manifest themselves at the macroscopic scale as photoluminescence (PL), while non-radiative events mostly transfer energy to the lattice vibration modes and thus manifest themselves at the macroscale via heat production.

Recombination efficiency is of course a very important parameter of a photocatalyst, as it correlates inversely with the photo-transformative efficiency. Therefore, techniques devoted to the study of recombination efficiency and timescales are of primary importance for TiO_2_ studies. It is well established that the only contribution to photoluminescence in a semiconductor that can occur at a relevant intensity is the band-to-band recombination in crystals with direct bandgap, where the opposite charge carriers can recombine directly in a first-order radiative transition, i.e., with emission of photons whose energy is approximately equal to the bandgap energy (with variability of the order of the thermal energy k_B_T) [166]. TiO_2_ has an indirect band-edge configuration and hence its PL emission occurs at wavelengths longer than the bandgap wavelength: that is, the PL of TiO_2_ is not caused by band-to-band transitions but involves localized states. As a consequence, it is easily understood why the TiO_2_ PL is typically quite weak and why it is ordinarily assumed that the vast majority of electron-hole recombination in this material occurs by non-radiative processes.

Achieving a direct detection of non-radiative recombination events is typically difficult, and actual estimations of non-radiative yields and lifetimes usually rely on indirect determination (such as for example evaluation of generated heat) [76].

On another hand, the radiative transition events can be directly detected by continuous-wave photoluminescence (CW-PL). Experimental setups suitable to compare in a quantitatively accurate manner the PL intensity from an investigated material and from a reference chemical species (e.g., a solution of some luminophore molecules) allows the determination of the PL quantum yield of the material under investigation. The is the quantity that more accurately (and directly) allows to estimate the actual proportion between radiative vs. non-radiative efficiency. The few available reports of PL quantum yield (η) measurements confirm this statement. In fact, typical values for η in various forms of TiO_2_ gives values of the order of ~0.1 to 1% [167,168,169,170,171] confirming the general idea that the vast majority of electron-hole recombination in TiO_2_ is non-radiative.

Analysis conducted by CW-PL are important also for other reasons. Consider for instance the Lambert–Beer law, which states that the amount of electromagnetic intensity I(z) which is not extinct via optical absorption after travelling inside the material for a length z is given by $I\left(z\right)=I_{0}exp\left[-\alpha \left(\omega \right)z\right]$, where α is the absorption coefficient. As typical values for the absorption coefficients at optical frequencies in TiO_2_ range in the order of ~10^5^ cm^−1^, we deduce that most of the photogenerated charges occurs within a region whose depth below the semiconductor surface has a typical value of few tenths of nanometers [172]. Therefore, the PL characteristics (e.g., spectrum, intensity, lifetime) are very sensitive to molecular adsorption and to surface chemical reactions, which are decisive factors for characterizing the behavior of the photocatalyst. As a consequence, since the previously-mentioned studies by Anpo and coworkers [161,162,163,164,165] PL has been regarded as an important tool for studying photocatalytic materials and surface properties [85,90,173,174,175,176,177,178,179,180,181,182].

A very peculiar characteristic of TiO_2_ PL is that its A and R phases exhibit different and well-separated bands, where anatase typically shows a broad emission in the visible spectrum (VIS-PL) peaked at ~500–530 nm while rutile shows a narrow spectrum peaked in the near-IR (NIR-PL, peaked at ~820 nm) [183]. The electronic processes and the nature of the different states responsible for such a difference are not completely elucidated.

#### 5.2.3. Anatase Photoluminescence

Regarding the origin of the anatase VIS-PL, two different hypotheses have gained greater consensus in recent decades, the first attributing is to the radiative recombination of self-trapped excitons and the second instead invoking free-to-bound radiative transitions, that is transition from defective (trap) states to band states or vice-versa.

Self-trapped excitons have been extensively invoked in early papers, based on the fact that the strong coupling between charges and crystal lattice field (i.e., electron-phonon interaction) typical of an ionic crystal can lead to a rearrangement of the local bond geometry associated to a relaxation of the exciton energy. The self-trapping of excitons is analogous to the self-localization of free charges as small polarons in ionic solids and has been observed in different alkali halides and large-bandgap oxides [184,185]. Some works interpreted the VIS-PL emission in anatase as the radiative dissociation of self-trapped excitons. According to this explanation, the more distorted Ti-O octahedral geometry of anatase (respect to rutile) favors the self-trapping of excitons, while instead excitons in rutile are free.

This interpretation leaves some question unanswered. For example, it is not clear why the emission originating from the recombination of free excitons in rutile is not observed and it is not explained why rutile emits light in the near-IR. Moreover, the self-trapped exciton interpretation seems problematic in relation to the more recent investigations on anatase PL emission under below-bandgap excitation (i.e., in conditions where the excitons cannot be formed) [186,187].

Successive works [83,90,188,189,190] instead argued in favor of the concurrence of two emission bands in anatase PL, caused by free-to-bound transitions (i.e., recombination of a free carrier and a trapped carrier). These transitions are typically centered at photon energies of (about) 2.4 and 1.9 eV [186], corresponding to respectively the green and the red region of the visible spectrum (hence hereafter named as “green” and “red” PL). These emission bands are observed simultaneously, so that they invariably give rose to the previously-mentioned broad “VIS-PL”, but careful studies of the interplay between these emission bands and the presence of external chemical species adsorbed on anatase surfaces conducted by the McHale group [83,182,191,192] and the Lettieri group [186,193,194] suggested the non-equivalence of these two PL contributions, i.e., that they are governed by different underlying mechanisms.

In this regard, Pallotti et al. evidenced the physical non-equivalence of the red and green PL via experiments on anatase/O_2_ (gas) interactions and by using PL excitation (PLE) spectroscopy technique [186]. In particular, PLE experiments allowed to show that the green component of the PL is dominant for supragap excitation ($\lambda_{exc}<387\;\mathrm{nm})$, while subgap excitation also induces a PL emission which is anyway shifted toward the red part of the spectrum.

A considerable number of findings [195,196,197,198,199,200,201,202,203,204] indicates that exposure to O_2_ quenches the PL intensity of a semiconductor: Such O_2_-induced quenching of the photoluminescence is quite naturally interpreted as the consequence of the scavenging of photogenerated free electrons in the CB (conduction band) operated by the oxygen molecules while adsorbing on the anatase surface. This charge-capture process is clearly competitive with the relaxation and/or radiative recombination of the photogenerated electron. Therefore, it quenches the PL emission that originates starting from free electrons.

**Figure 13:**
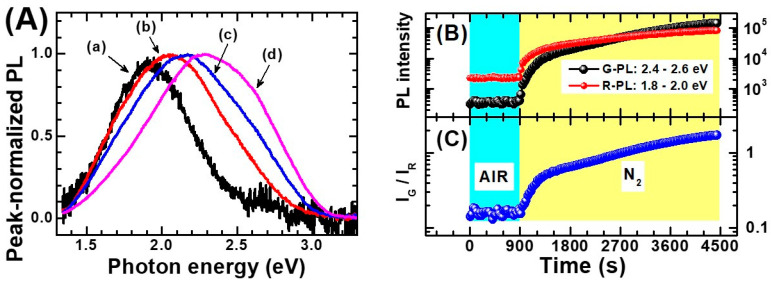
(**A**) Peak-normalized photoluminescence (PL) spectra of commercial anatase NPs measured after 900 s exposure to dry air (curve (a)) and after 600 s, 1500 s and 3600 s (curves (b), (c) and (d), respectively) of exposure to dry N_2_ flow. The progressive shift toward shorted wavelengths of the PL spectrum indicates that the quenching effect exerted by O_2_ is more pronounced on the “green” component (G-PL) of the photoluminescence spectrum of anatase. (**B**) PL intensity vs. time integrated in the photon energy intervals of 2.4–2.6 eV (G-PL, black dots) and 1.8–2.0 eV (R-PL, red dots). (**C**) Ratio between the G-PL and R-PL intensities vs. time. The increase of this ratio is another way to indicate the larger sensitivity of the green PL to the O_2_ desorption. The exposure to different gaseous atmospheres is represented by differently colored backgrounds: Cyan: air, yellow: N_2_. G-PL and R-PL state for green PL and red PL, respectively.

The O_2_-induced quenching of PL is easily observed in anatase TiO_2_ and is particularly prominent for the “high energy side” (i.e., green PL) of the emission spectrum. An example of this is shown in Figure 13.

**Figure 14:**
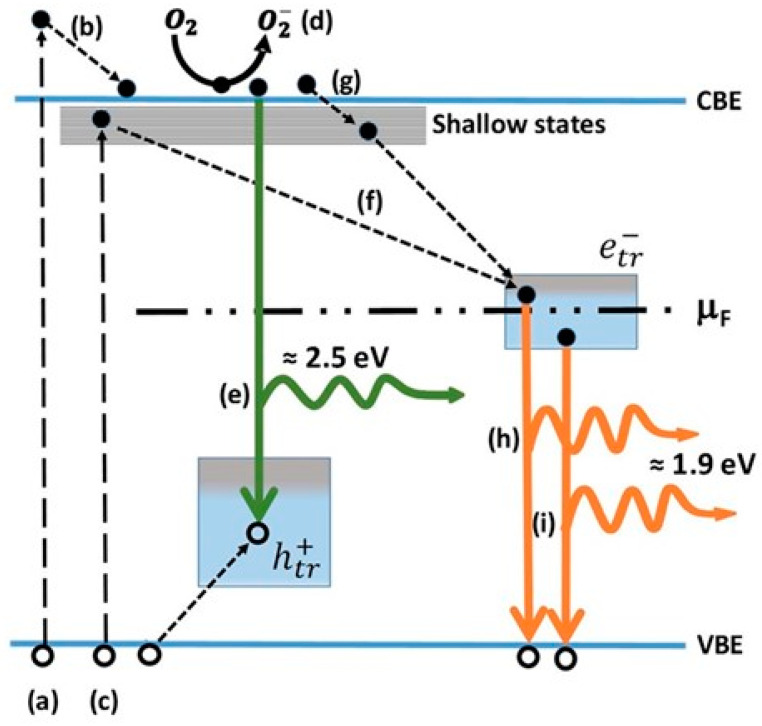
Schematic of the anatase PL mechanisms: (**a**) photo-excitation of charge carriers (electrons and holes) via absorption of UV light; (**b**) electron relaxation (thermalization) in the conduction band; (**c**) excitation of band-tail states (shallow states) via absorption of below-bandgap light; (**d**) scavenging of free electrons by O_2_; (**e**) radiative decay of conduction band electrons in electron traps with emission of “green” PL; (**f**,**g**) non-radiative decay of donor-like electrons (from shallow states) and free electrons (from the conduction band) to trap states; (**h**,**i**) radiative decay of trapped electrons to valence band, with emission of “red” PL peaked at about 1.9 eV. In this scheme this latter emission can be caused by either radiative recombination of electron in trap states to the valence band (mechanism “h”) or by radiative recombination of photogenerated holes with electrons in trap states below the Fermi level μ_F_ (these latter states can be already occupied before the laser excitation). Reprinted with permission from Reference [186]. Copyright 2017 American Chemical Society.

**Figure 15:**
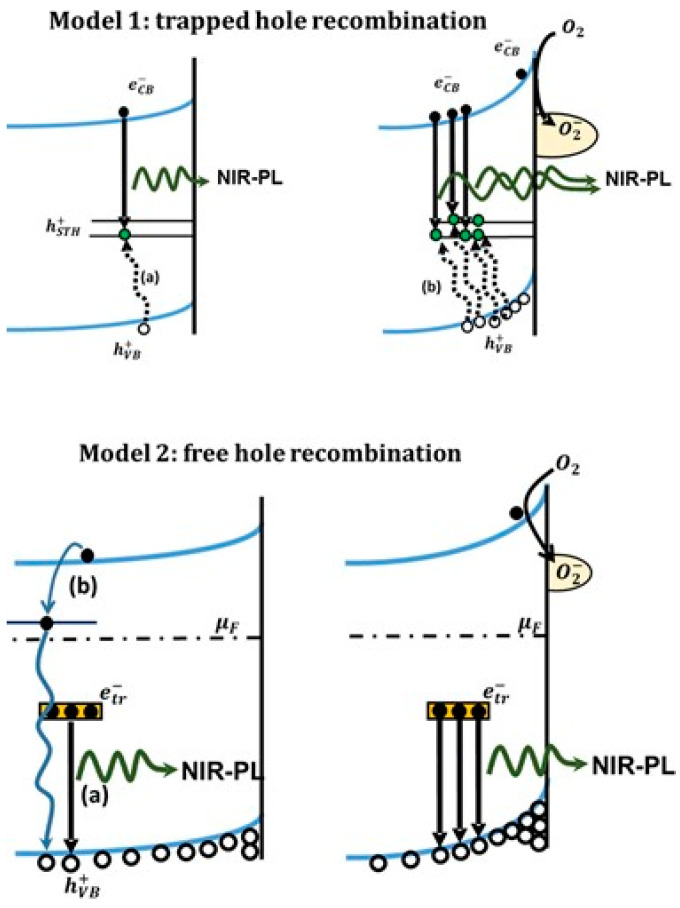
The “Model 1” (scheme on top of the Figure) is based on the hypothesis that the rutile PL is initiated by a self-trapped photogenerated hole close to a 3-fold coordinated O atom, which gains energy with respect to bulk holes and then recombines radiatively with a conduction band electron. This mechanism could explain the enhancement of the rutile PL caused by O_2_ adsorption, as the adsorbed superoxide ions bends upward the valence band and then helps accumulating holes near the surface. In the scheme below (“Model 2”) the near-infrared PL of rutile instead involves free holes and electrons trapped in mid-gap states below the Fermi level (which are already occupied in dark conditions), as shown by mechanism “a” in lower left. In this case, the enhancement of near-IR PL caused by O_2_ can be interpreted as the consequence of a different competition for the recombination of the photogenerated holes in the valence band. In presence of O_2_ more free electrons are scavenged (while those already in the trap states in dark condition are not affected by the adsorbed O_2_) and thus there is less competition for the recombination with the photogenerated holes. Reprinted with permission from Reference [186]. Copyright 2017 American Chemical Society.

Based on the beforementioned studies by the McHale group and Lettieri group on TiO_2_ photoluminescence, a scheme for the totality of recombination processes in anatase and rutile can be proposed and schematically shown in Figure 14 for anatase (for additional details see Reference [186]). It has to be underlined that, at the present date, a global consensus on the overall configuration of processes that are involved in TiO_2_ PL is still not present. Hence, the detailed mechanisms discussed in caption of the Figure 14 and Figure 15 shall therefore be considered as reasonable hypothesis, even if most of the argumentations appear to be pretty solid.

The following points regarding the A-TiO_2_ photoluminescence appear to be most experimentally established and the soundest from an interpretative point of view:

(1) Non-equivalent recombination processes characterize anatase PL and lead to two spectrally separated contributions in the “green” and in the “red” part of the visible spectrum (i.e., centered at about 520 and 650 nm).
(2) The overall anatase PL (i.e., both their spectral contribution) are subject to intensity quenching when the surface is exposed to O_2_. However, the “green” contribution is more sensitive than the “red”.
(3) The excitation spectrum of anatase TiO_2_ follows the interband absorption curve. However, additional PL emission is still present at sub-bandgap excitation conditions [188], in particular when the titania has been subjected to reductive treatments [207]. This evidence indicates that the anatase PL is not exclusively caused by self-trapped exciton recombination, as stated in several earlier papers (this point has been mentioned at the beginning of the present subsection)

Based on the above points, a mechanistic picture of the PL processes of A-TiO_2_ can be proposed as represented in the next figure, involving two population of trapped charge-carriers. The first consists of trapped holes that can recombine with free electrons. Given that this transition is the one sensitive to electron scavengers and based on the available data on O_2_-induced quenching of A-TiO_2_ PL, the same transition is likely to be associated to the green emission.

The second population of trapped charges that hence shall be assigned to the “red PL” and consists of trapped electrons which are deeply trapped and hence less likely to be chemisorbed by O_2_. However, some PL quenching is also expected due the development of a depletion region (see Section 5.3.1 for a discussion on this point). Moreover, some of the trapped electrons may originate from photogenerated electrons (see processes “f” and “g” in Figure 15) and they can be chemisorbed due to O_2_ exposure.

#### 5.2.4. Rutile Photoluminescence

Interpretating the origin of the rutile NIR-PL is more difficult, for the reasons discussed here. Before starting the discussion, we point out the evidence that appear to be experimentally established:

(1) Rutile NIR-PL occurs at with a peak energy typically included in the wavelength interval 820–850 nm. While room temperature measurements typically display a single peak, some reports resolve different contributions (for example Reference [206])
(2) The PLE analyses indicate that the rutile PL is clearly initiated by free carriers only, as no NIR-PL emission at sub-gap excitation is observed.
(3) Exposing the rutile to O_2_ enhances its PL (contrary to the case of anatase).

Concerning the NIR-PL emission of rutile, a general scheme that has been carried forward in several works by Y. Nakata and coworkers [207,208,209] attributes the NIR-PL to the decay of conduction electron with “self-trapped holes”, as schematized as “model 1” in Figure 15.

Another interpretation regarding the origin of the NIR-PL was proposed by Santara and coworkers [210], who stated that it has to attributed to recombination of electrons trapped at interstitial Ti sites close to the surface. The authors based this statement on PL studies of intrinsic mixed-phase TiO_2_ nanoribbons (exhibiting different mixed phases, depending on the temperature of the solvothermal assembling process), corroborated by EPR and XPS analyses. It is to be noted that the attribution of NIR-PL to recombination of electrons trapped near interstitial Ti atoms appears to be incompatible with the before-mentioned “self-trapped holes” scheme.

The same authors suggested that the NIR-PL is not an intrinsic property rutile phase only. Instead, it might be simply observed prevalently in rutile because the rutile phase is usually produced by carrying out a heat treatment of anatase phase that conceivably activates the migration of intrinsic defects from the bulk to the surface. Among them, the interstitial Ti sites (including both Ti^4+^ and Ti^3+^) also migrate toward the surface and their larger surface density (in comparison to the anatase one) might account for the NIR-PL.

Finally, it is worth noting that NIR-PL emission has been evidenced also in the TiO_2_(B) phase (brookite titania) as shown by Vequizo and coworkers [210]. In the latter work, the authors observed that methanol vapor quenched both the visible and NIR emissions of TiO_2_(B) due to the hole-consuming reaction of methanol (a known hole scavenger), while O_2_ quenched the visible emission and enhanced the NIR emission, as also observed (as previously mentioned) for anatase and rutile phases. The authors interpreted the data stating that the NIR-PL is caused by the radiative recombination of electrons deeply trapped in surface sites at energies close to the mid-gap level and valence band holes.

It is to be noted that in Reference [210] the chemical nature of the sites that trap deeply the electrons is not specified, but this hypothesis looks compatible with that of Reference [206] which assign these sites to interstitial Ti.

### 5.3. Interplay between Photogenerated Charges and Molecular O_2_ Adsorption: The O_2_-Dependent PL 

In this section we underline the peculiar phenomena occurring to PL intensity of TiO_2_ exposed to molecular oxygen (O_2_), as evidenced in relatively recent studies. The main feature here appears to be that TiO_2_ has not a unique response to O_2_ adsorption. More precisely: exposure to O_2_ can induce either PL enhancement or PL quenching in anatase, while several reports (as discussed previously) also indicate that O_2_ induces PL enhancement in rutile near-infrared (NIR) PL.

In most cases, O_2_ act as a reversible scavenger of photoexcited electrons, causing a PL decrease (quenching) that can be caused by two concurrent effects, namely (i): the trapping of charges which are hence no more available to the radiative recombination (see for example Figure 14), and (ii): an increased upward bending of the energy bands below the surface, causing an enlargement of electron-depleted region and hence a decrease in the spatial overlap between holes and electrons, ultimately leading to a lesser rate for their recombination within the excitation volume. Therefore, it is—generally speaking—quite unusual and puzzling to observe an O_2_-induced enhancement of PL.

#### 5.3.1. O_2_—Anatase Interaction

The interplay between anatase PL and O_2_ adsorption was studied in detail by Ma and coworkers [211], who proposed that the occurrence of either PL enhancement or PL quenching are basically related to opposite modifications of the surface potential (or, equivalently, the band bending toward the surface). As mentioned, the regions where upward band bending occur are partially depleted of mobile electrons and hence characterized by a lesser degree of spatial overlap between opposite charge carriers which is a necessary condition for the radiative recombination to occur. Hence, that any increase in the band bending is associated to a decrease in the region that can produce photoluminescence.

**Figure 16:**
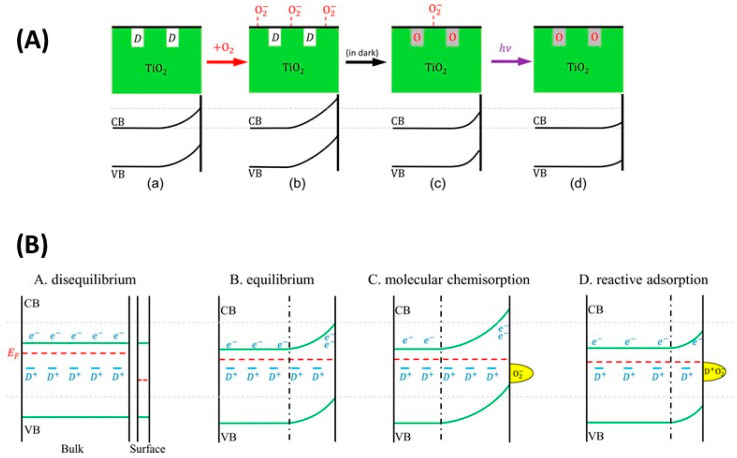
(**A**) Scheme for the two possible modification of the surface band bending of TiO_2_ induced by exposure to O_2_. The configurations (b) and (d) are associated to the lowest and highest PL intensity, respectively. (**B**) Interplay between adsorbed O_2_ and intrinsic defects of TiO_2_ as proposed by Ma and coworkers as the processes capable to modulate the PL intensity. The configuration shown in scheme D (labelled as “reactive adsorption”) is characterized by the presence of a neutral species, resulting from reaction between molecular oxygen and surface donor-type defects. According to the data shown in Figure 16, this latter mechanism is active only for low O_2_ exposures. Reprinted with permission from Reference [211]. Copyright 2017 American Chemical Society.

The fact that O_2_ could alter the TiO_2_ band bending in two different ways was attributed to the occurrence of two possible O_2_ adsorption processes, one characterized by O_2_/defect reactions leading to a drop in the surface potential (and PL enhancement) and another in which oxygen remains as chemisorbed superoxide ions on the surface, increasing the band bending and decreasing the PL intensity. Such a double possibility is schematized in Figure 16. Figure 16A shows the possible dynamics of the band bending, increasing due to the presence of chemisorbed charged molecular species (scheme b) and decreasing if due a reaction between molecular charged species and defects occurs in dark (scheme c), eventually followed by further lowering of the band bending and hence to increase of the PL intensity in by UV excitation in vacuum (scheme d).

Figure 16B (lower figure) also shows a possible interpretation of the actual process that leads to the variations of the band bending, involving an oxygen-defect (D^+^ in the scheme) reactive adsorption active reaction that lowers the surface charge and hence the band bending [211].

**Figure 17:**
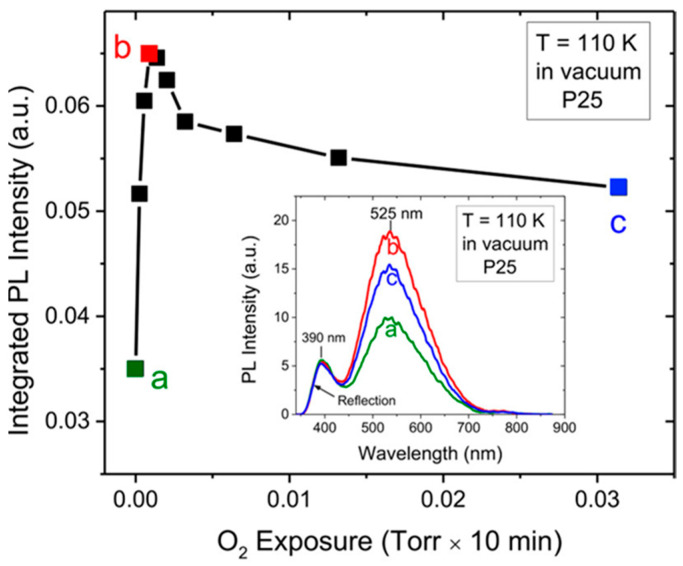
Effect of O_2_ exposure on the PL of P25 TiO_2_. The spectra were measured in post-exposure mode, that is: the TiO_2_ sample in the measurement chamber was exposed for 10 min to a pre-selected O_2_ pressure in dark, followed by chamber evacuation for 5 min and finally by acquisition of the PL spectra (obtained for 320 nm excitation wavelength). The spectra shown in the inset correspond to PL spectra acquired after a 10 min exposure to O_2_ pressures of (a): 0 Torr (i.e., high vacuum), (b): 9 × 10^−4^ Torr and (c): 3 × 10^−2^ Torr. All spectra were acquired at chamber temperature of 110 K. Reprinted with permission from Reference Reprinted with permission from Reference [211]. Copyright 2017 American Chemical Society.

An example of opposite behavior induced by O_2_ is shown in Figure 17 where the PL in vacuum conditions is measured after exposures at different O_2_ pressures. The data were interpreted by hypothesizing that the O_2_ adsorption at low exposures preferentially occurred via reaction with surface defects (schematized Figure 16b as “reactive adsorption”), thus leading to the observed PL increase (regions a,b in the Figure 17). On the contrary, at increasing O_2_ exposure the population of available defects decreased and the reactions with defects were saturated, so that the additional O_2_ occurred as chemisorbed $O_{2}^{-}$ ions causing the decrease of PL intensity (observed from point b to point c in Figure 17).

#### 5.3.2. O_2_—Rutile Interaction

As mentioned, one of the first interpretation for the origin of NIR-PL emission of rutile involved the idea of a radiative recombination between conduction electrons and “self-trapped holes” [207,208,209], also schematized as “model 1” in Figure 15. This model has a problematic element in the fact that, due to its electron-scavenging activity, adsorbed O_2_ favors the accumulation of mobile holes toward the surface and (equivalently) “pushes” mobile electron towards the bulk (hence increase the upward band-bending at the surface, as also shown in Figure 16). Therefore, the “self-trapped hole” model is compatible with the observed NIR-PL enhancement caused by O_2_ only if these trapped holes have a low mobility and are present also extend also in the bulk. However, this is a problematic point as the self-trapped holes are believed to be formed mainly at the rutile surface [207,208,209].

We now consider the model that attributes NIR-PL to interstitial Ti defects: is it compatible with the observed NIR-PL enhancement by O_2_ exposure? Concerning this point, Santara et al. [206] pointed out that some reoxidation experiments on reduced rutile TiO_2_ crystals exposed to O_2_ at high temperature evidenced the surfacing of Ti interstitial that were previously buried below the surface [212]. This phenomenon was suggested as an interpretation of NIR-PL enhancement under O_2_ exposure, once assumed that surface interstitial Ti species cause the NIR PL.

However, it can be argued that this interpretation is problematic in the sense that surfacing of Ti species is likely to be caused by the temperature increase and not by the exposure to O_2_ itself. As a matter of fact, the reoxidation process described by Onishi and coworkers [212] in which partially reduced Ti^3+^ ions accumulated in interstitial positions were oxidized at the surface has been observed for surfaces heated at 800 K, while all the experimental evidence indicate that NIR-PL enhancement of rutile (and of brookite, see Reference [210]) occurs at room temperature.

It is our opinion that the attribution of NIR-PL to surface Ti interstitial defects is not problematic in itself, as the enhancement in O_2_ might occur simply because the adsorbed O_2_ accumulates valence band holes close to the surface and then increase the overlap between the recombining charges. However, to the best of our knowledge it is still not completely clear why the same defects are not found in anatase phase. Additional in-depth analysis is probably still necessary to answer the question about the physical origin of the NIR-PL.

#### 5.3.3. Applications of O_2_-Dependent PL of TiO_2_

In principle, the sensitivity of TiO_2_ PL to molecular oxygen could be employed for oxygen sensing by detecting the modification of PL intensity. However, it has to be underlined that any detection approach based on the measurement of luminescence intensity is liable to errors. In fact, an absolute measurement of PL intensity is by definition not expressed in comparison to a proper reference value which might be for example the excitation intensity or—equivalently—any “secondary” intensity signal proportional to the excitation intensity. As a consequence, fluctuations in the intensity of excitation light will introduce an uncertainty in the experimental data which can be troublesome (in particular if the intention is to use the system to perform real-time monitoring).

One way to circumvent this technical issue involves the use of a ratiometric detection, i.e., detecting two PL intensities, both proportional to the excitation intensity but one being insensitive to the analyte while the second being sensitive to it. Hence, the ratio between the two signals will cancel out uncertainties caused only by a fluctuation in the excitation intensity.

It is interesting to mention that a similar concept can be implemented for mixed phase TiO_2_ due to the fact that the PL of the rutile and of the anatase forms react differently to O_2_ exposure under UV illumination conditions. This peculiarity of TiO_2_ has been indeed exploited in Reference [213], showing that by using a mixture of rutile and anatase nanoparticles the sensitivity of the O_2_-induced PL changes can be increased significantly—in comparison with the one obtainable by single-phase nanopowders—using the ratio (rutile PL/anatase PL) as the parameter that signal the presence of O_2_. That leads to a “ratiometric” responsivity:

(4) $$R=1-\frac{\phi_{NIR}^{0}}{\phi_{NIR}}\frac{\phi_{VIS}^{0}}{\phi_{VIS}}$$

which is by construction larger than the one obtainable using single-phase titania (for the demonstration see Reference [213]). In Equation (14) “NIR” and “VIS” refer to the near-infrared PL of rutile and to the visible PL of anatase (respectively), while $\phi^{0}$ and ϕ are the PL intensity in absence and in presence of O_2_, respectively.

**Figure 18:**
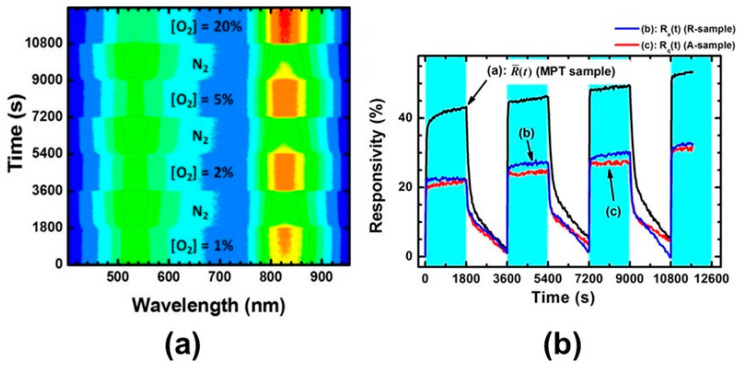
(**a**) Contour plot of PL spectral intensity measured during the exposure to oxygen/nitrogen mixtures with different O_2_ concentrations. (**b**) Time-dependent optical responsivities R(t) at different O_2_ concentrations for the mixed-phase sample (“mixed phase titania”, labelled as MPT) corresponding to the data shown in (**a**) and for two single-phase samples (anatase and rutile nanopowders). The responsivity for the mixed-phase sample is indicated in Equation (14), while the expression for the responsivities for anatase (showing a quenching response Rq) and rutile (showing an enhancement response RE) are reported in Reference [213]. The O_2_ concentration values are reported in (**a**). Reprinted from Reference [213], with the permission of AIP Publishing. Copyright 2016 AIP Publishing.

An example of this concept is shown in Figure 18, that shows the dynamical (i.e., time-dependent) PL response of the mixed-phase titania toward different O_2_ pressures under UV excitation at 325 nm. The graphs evidence the anti-correlated behavior of the two PL bands. From the PL curves, the effective response ratiometric response defined by Equation (4) and obtained from the data shown in Figure 18a are reported in Figure 18b and compared to the response curves which are obtained from single-phase titania.

**Figure 19:**
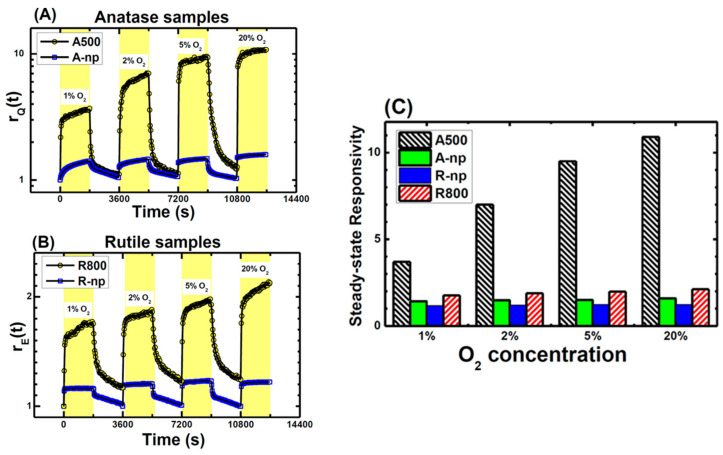
(**A**) Responsivity of hierarchical 1D anatase film (black curve, quenching response) as exposed to variable concentrations of O_2_. The black curve is compared to the data obtained in the same experimental conditions for commercial anatase nanopowders (blue curve). It shall be noted that the vertical scale has logarithmic units. (**B**) Responsivity of rutile film obtained by thermal annealing of the hierarchical 1D anatase film. As the TiO_2_ nanoparticles coalesce at the anatase-rutile transition, the specific surface area decreases and so the gas responsivity. Nevertheless, the response (black curve) was found to be larger than the one exhibited by commercial rutile nanoparticles (blue curve). In both (A) and (B) the exposure to O_2_ takes place in the yellow-colored time intervals, followed by exposure to flowing nitrogen (white regions). The tested oxygen concentrations (in percent units) were 1%, 2%, 5% and 20%. (**C**) Steady-state maximum responsivities of the tested samples measured for different O_2_ concentrations. Adapted with permission from Reference [34]. Copyright 2017 American Chemical Society.

It is worth underlining that the modulation of TiO_2_ PL intensity can be very large, relatively to the typical values that are usually observed for other metal oxides exposed to gas analytes. As an example, Reference [34] reported changes in PL intensity in hierarchical anatase-phase titania [151] with responsivities up to about 1100% at 20% O_2_ concentrations, which outperformed those obtainable by commercial TiO_2_ nanopowders up to a factor of about 7 for response to synthetic air (see Figure 19).

These values are remarkable, when compared to some of the experimental findings reported in literature on PL-based opto-chemical gas sensing using other metal oxides (see Reference [34] and supporting information therein).

## 6. Intrinsic TiO_2_ as Photocatalyst: Mechanisms and Limits

### 6.1. Basic Photocatalytic Processes and Their Characteristic Times 

**Figure 20:**
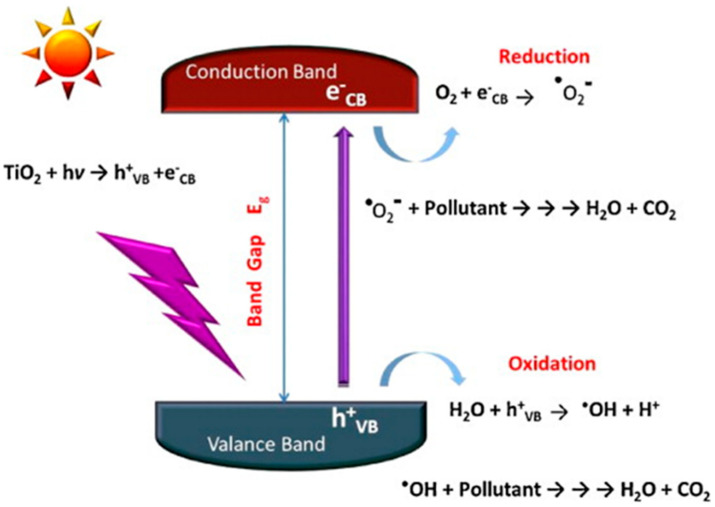
Graphic representation of the main processes that occur after the photogeneration of charge carriers in undoped TiO_2_. Reprinted with permission from Reference [214]. Copyright 2105 Elsevier Science.

As mentioned, the photo-induced degradation of pollutants using a semiconductor photocatalyst involves the absorption of photons whose quantum energy ℏω=hc/λ is sufficient to generate free charges (that is, electrons in the conduction band and holes in the valence band of the semiconductor) capable to migrate toward the surface and, once there, to participate in the oxidation-reduction reactions with suitable chemical species. These latter species can eventually be the pollutant themselves that will be transformed via direct photodegradation. However, it is more likely that the mentioned redox reactions will involve the dissolved oxygen (O_2_) and/or the hydroxide ions ($OH^{-}$) present in aqueous environment, with formation of reactive oxygen species (ROS) that, in turn, will decompose the pollutants. A graphical representation of the main photocatalytic processes that occur after the photogeneration of charge carriers is shown in Figure 20.

According to the prevailing understanding of photocatalytic phenomena, the most active species generated by illumination of TiO_2_ in aqueous environment are the superoxide radicals ($O_{2}^{•-}$), the hydroxyl radicals ($HO^{•}$) and the photogenerated holes ($h^{+}$). The superoxide radicals are generated from the dissolved molecular oxygen naturally present in the aqueous solution via photoreduction by a conduction band electron:

(5) $$e_{CB}^{-}+{\left(O_{2}\right)}_{\left(gas\right)}\to {\left(O_{2}^{•-}\right)}_{\left(ads\right)}$$

The hydroxyl radicals represent the primary oxidant in the photodegradation processes and are typically generated via two routes:

(1) $OH^{-}$ ions in water are oxidized by photogenerated holes (see Equation (6)).
(2) Superoxide radicals react with protons ($H^{+}$) forming peroxidic radicals ($HOO^{•}$), which, in turn form oxygen peroxide that further decomposes to hydroxyl radicals (see Equations (7)–(9)). Finally, photogenerated holes can also oxidize a pollutant molecule directly and trigger its degradation (see Equation (8) next). The corresponding reactions are:
  (6) $$h^{+}+OH^{-}\to HO^{•}$$
  (7) $$H^{+}+O_{2}^{•-}\to HOO^{•}$$
  (8) $$2HOO^{•}\to O_{2}+H_{2}O_{2}$$
  (9) $$H_{2}O_{2}+O_{2}^{•-}\to O_{2}+OH^{-}+HO^{•}$$
  (10) $$h^{+}+\left(polluttant\right)\to {\left(polluttant\right)}^{+}$$

**Figure 21:**
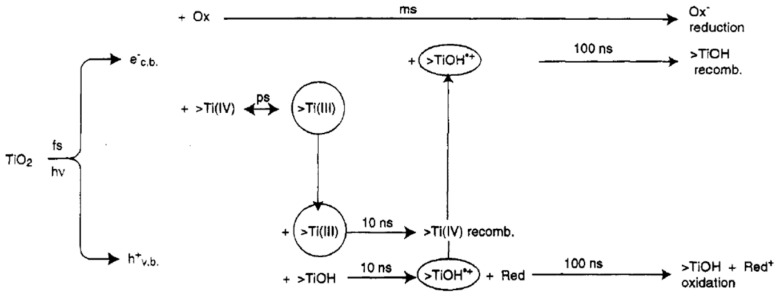
Schematic representation of the recombination processes of the photo-excited charge carriers in TiO_2_. The arrow length of each process is representative of its time scale. Reprinted with permission from Reference [84]. Copyright 1995 American Chemical Society.

A review by Hoffman, Bahnemann and coworkers [84] summarized the relevant photocatalytic processes of photoexcited TiO_2_ and their time dynamics, based on the results of investigations conducted via time-resolved microwave conductivity experiments [215,216]. The dynamical scheme produced by Hoffmann et al. (also referenced also in other successive reviews) [19,217] is shown in Figure 21 and summarized as follows. For a time duration of about 10 ns after the photogeneration of an electron-hole pair, mobile electrons ($e_{CB}^{-}$) can be trapped at shallow or deep levels localized on Ti^4+^ sites, while mobile holes ($h_{VB}^{+}$) can be trapped on hydrated surface functionalities (TiOH), forming surface-bond hydroxyl radicals, here indicated by ${\left(TiOH^{•}\right)}^{+}$ using the nomenclature of Ref. [84]. On the same time scale, the trapped electrons can recombine with mobile holes. These processes can be represented as follows (we also indicate the characteristic time scale for each one):

(11) $$e_{CB}^{-}+Ti^{4+}\to Ti^{3+}\;(\mathrm{Shallow}\;\mathrm{traps}:\;<1\;\mathrm{ns}.\;\mathrm{Deep}\;\mathrm{traps}:\;\sim 10\;\mathrm{ns})$$

(12) $$TiOH+h_{VB}^{+}\to {\left(TiOH^{•}\right)}^{+}\;(\mathrm{Surface}\text{-}\mathrm{trapped}\;\mathrm{holes},\;\sim 10\;\mathrm{ns})$$

(13) $$h_{VB}^{+}+Ti^{3+}\to Ti^{4+}\;(\mathrm{Recombination},\;\sim 10\;\mathrm{ns})$$

Hence, both the charge localization and (non-radiative) recombination is likely to occur on typical scales of about 10 ns, after which—according to the model by Hoffman et al.—the surviving charge are mainly surface-trapped holes localized at Ti-OH terminations. On successive stage, these holes can recombine with mobile electrons (Equation (12)) or oxidize an external species Red (see Equation (13)). On another hand, the same authors proposed slower dynamics for typical oxidation processes by photoelectrons (Equation (13)), i.e.,

(14) $${\left(TiOH^{•}\right)}^{+}+\;e_{CB}^{-}\to TiOH\;(\mathrm{Recombination},\;\sim 100\;\mathrm{ns})$$

(15) $${\left(TiOH^{•}\right)}^{+}+\;Red\to TiOH\;+{\left(Red\right)}^{+}\;(\mathrm{Oxidation},\;\sim 100\;\mathrm{ns})$$

(16) $$e_{tr}^{-}+Ox\to {\left(Ox\right)}^{-}\;(\mathrm{Reduction},\;\sim \mathrm{ms})$$

There is clearly a competition between charge recombination and photocatalytic transformations. This competition is clearly in favor of the former, as long as the redox lifetimes vary in a range from hundreds of nanoseconds to hundreds of microseconds, while the recombination occurs with typical lifetimes of 1–10 ns.

In this model, it is argued that only the trapped charges do participate to the redox processes and that there is no direct oxidation by the conduction band holes. Indeed, as mentioned previously, the photogenerated holes are nowadays also considered possible oxidant for a direct degradation of organic contaminants [218]. However, whichever the case, a kinetic competition between the recombination of the charges (either trapped or mobile) and the redox reactions always occurs and the recombination rate is always a primary limiting factor of the photocatalytic efficiency. This observation justifies the importance of time-resolved studies of charge dynamics in TiO_2_ and other metal oxide photocatalysts under operating conditions [173,195,219].

According to the model by Hoffman and coworkers, the O_2_ photoreduction is the slow reaction and thus the limiting step of TiO_2_ photocatalytic activity in aqueous solutions. In fact, if the O_2_ reduction is not fast enough to match the rate of reaction of holes, an excess of electrons will accumulate on the catalyst and the rate of electron-hole recombination will increase, limiting the photocatalytic efficiency. This was indeed proved to be the case by Gerischer and coworkers [220,221] who proved that an excess of trapped negative charge at TiO_2_ surface can persist for about 1 min even in saturated O_2_ solutions, unless the electrons are consumed by some other additional species more prone to be reduced (e.g., Pt).

The lesser tendency to oxygen reduction (compared to hole-induced oxidation) has consequences on the formation of oxygen peroxide, as the presence of dissolved O_2_ was proved to be necessary for the formation of oxygen peroxide (see Equations (7) and (8) by employing isotope-labeled (^18^O) study of oxygen photoreduction, showing that all of the H_2_O_2_ arises from $O_{2}^{•-}$ generated via conduction band electrons (see Equation (5)) [222].

Hydroxyl radicals are widely regarded as the primary and most active oxidant in TiO_2_ based photocatalysis. Evidences supporting this idea were reported in experiments on photocatalytic degradation of halogenated aromatic species, showing that the degradation intermediates mainly consist of hydroxylated structures, consistently to what is observed when similar aromatics react with known sources of $HO^{•}$ radicals [223,224,225,226]. Furthermore, the presence of hydroxyl and hydroperoxyl radicals was also proved by electron paramagnetic resonance spectroscopy [227]. In more detail, the abstraction of H atoms by the hydroxyl radicals has been evidenced to be a primary oxidation step that determines the overall photodegradation, as highlighted for example by studies showing that the oxidation chlorinated ethane correlates with the C-H bond strengths of the reactant [228].

On another hand, several other published findings indicate that the hydroxyl radical is not necessarily the only species responsible for photocatalytic activity. To this regard it is worth mentioning that cases have been reported in which the reactants had no H atoms available for abstraction by $HO^{•}$ [228,229] and in which the photocatalytic transformation has been attributed to a direct hole transfer [230].

In conclusion, different species can concur simultaneously to specific photocatalyzed transformations. More detailed information on the activity of a given oxidizing species can be obtained dissolving in the aqueous solution species that quench the activity of a specific reactive radical [231].

### 6.2. Limitations of Intrinsic TiO_2_ as Photocatalyst

The present and near-future trends in TiO_2_ based photocatalytic technologies are fundamentally influenced by the limitations of intrinsic TiO_2_, the most important being (A): its inability to be activated by visible light (e.g., sunlight), and (B): the scarce charge separation.

We first consider the problem of visible light-induced activation. As mentioned previously, the band gap energy of anatase TiO_2_ (the most photocatalytically active polymorph) is about 3.2 eV, which—using the relation in Equation (1)—corresponds to photons of wavelength $\lambda_{g}=387\;\mathrm{nm}$ lying in the near ultraviolet (or “UV-A”) interval of the electromagnetic spectrum. Unfortunately, this implies that TiO_2_ is scarcely activated by solar radiation, as the near-UV light accounts for just about 5% of the total solar energy on Earth’s surface.

The second point is the scarce charge separation. Larger proximity of electrons and holes imply larger recombination rates and lower lifetimes of the photogenerated charge carriers, so that most of them will recombine before reaching the surface where the photocatalytic processes (such as ROS generation) shall occur.

In order to overcome these problems, several strategies have been scrutinized. Here, we will schematically classify them in two main categories, namely:

(1) Strategies based on engineered TiO_2_ nanocrystals, that is centered on modifications and/or control of intrinsic properties of TiO_2_ nanocrystals, such as the stoichiometric composition or the surface termination (“facet engineering”),
(2) Strategies based on the use of heterojunction photocatalysts, i.e., composites materials or in which TiO_2_ is electronically coupled with a different material acting as cocatalyst.

We discuss separately these two categories in Section 7.1 and 7.2. Engineered TiO_2_ nanocrystals are discussed in Section 7.1, with specific focus on highly-reduced TiO_2_ (black and “colored” TiO_2_) and on the exploitation faceted TiO_2_ nanocrystals, based on the natural tendency of electrons and holes to localize on different crystal termination in anatase.

The second category (Section 7.2) deals with some of the most used heterojunction photocatalysts and co-catalysts. The intrinsic length limitations of the present review will not allow a complete review of these extensively-studied topics. Aspects regarding the “hottest” topics will be discussed next. The reader is referred to other more specialized reviews for further information [2,19,21,22,232,233,234,235].

## 7. Present and Future Trends for TiO_2_-Based Heterostructure Photocatalysts and Engineered TiO_2_

### 7.1. Engineered TiO_2_ Nanocrystals

The approaches described in the section do not recur to the use of cocatalysts. In particular, we consider here the strategies based on highly reduced TiO_2_—also often referred to as “black TiO_2_” or more generally to “colored TiO_2_”—and on facet engineered nanocrystals, in which the key role is played by the control of exposed crystalline facets.

#### 7.1.1. Black TiO_2_

The preparation and study of TiO_2_ nanocrystals having intermediate to high levels of oxygen reduction is currently regarded as one of the most interesting approaches among that not relying on the use of cocatalysts. The concentration of oxygen vacancies is directly correlated with the optical absorption spectrum and hence on the apparent color of TiO_2−x_ nanoparticle aggregates. Samples with very high concentrations of oxygen vacancies appear as dark grey or even black as the result of a large and approximately wavelength-independent optical absorption in the visible spectrum. These samples are referred to in literature as “black TiO_2_” [22,236,237,238] or as “grey” or otherwise “colored” version of TiO_2_ (e.g., blue TiO_2_) [205,239,240]. In all of these cases, the formation of defects which are not present in pristine TiO_2_ nanoparticles leads to an additional optical absorption band (“additional” here meaning that is absent in stoichiometric TiO_2_) and then to the sample coloration. The additional optical absorption band is associated to optical transitions that involve electrons trapped in the vacancy sites [104,233] and permits the formation of photogenerated carriers even under illumination with visible light, which is the general goal of this kind of approach.

**Figure 22:**
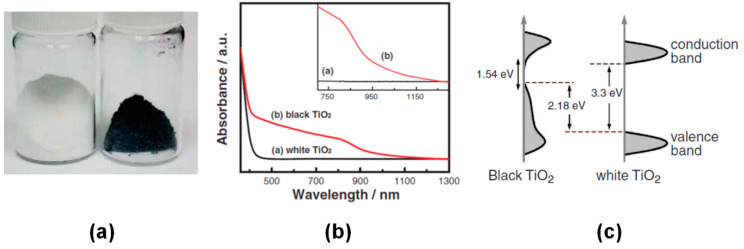
(**a**) White and black TiO_2_ powders. (**b**) Optical absorption spectra of white TiO_2_ and black TiO_2_, evidencing the slowly-varying non-zero optical absorption in the visible range, which extends even in the near-infrared range (evidenced in the inset). (**c**) Scheme of the electronic density of states of Black TiO_2_ (compared to standard “white” TiO_2_), showing almost no optical bandgap due to the extended tail of states responsible of the black coloration. Reproduced with permission from Reference [236]. Copyright 2011, American Association for the Advancement of Science.

Earlier evidence of the possibility to obtain “black TiO_2_” via hydrogenation process at high temperatures was reported by Chen and coworkers in 2011 [236], who demonstrated that the introduction of a disordered surface layer via hydrogenation led to formation of extended “band-tail” states (see Figure 22c) responsible for the enhanced optical absorption of visible light (see Figure 22b). The black TiO_2_ powders also exhibited effective photocatalytic properties, including photodegradation of the methylene blue dye and H_2_ production [236]. The pioneering work of Chen and coworkers determined a huge interest in black and, more generally, in colored versions of TiO_2_, such as “grey” and “blue” titania.

**Figure 23:**
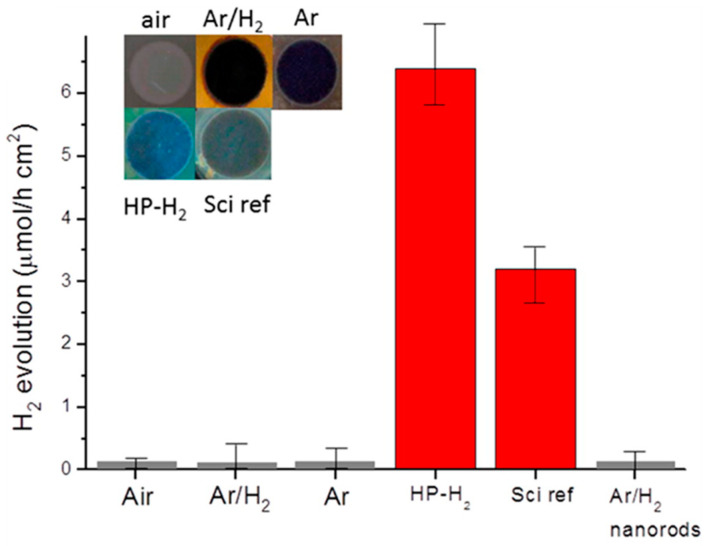
Rate of photocatalytic H_2_ production in 50/50% volume water/methanol solution under AM1.5 illumination and in open circuit conditions by using TiO_2_ nanorods and nanotubes treated in different atmospheres. Air: heat treatment in air at 450 °C. Ar: heat treatment in pure argon at 500 °C. Ar/H_2_: heat treatment in H_2_/Ar (5 vol%) at 500 °C. HP-H_2_: heat treatment in H_2_ at 20 bar at 500 °C. Sci ref: heat treatment in H_2_ at 20 bar at 200 °C for 5 days. Inset: optical images for the differently treated samples. Reprinted with permission from Reference [243]. Copyright 2014 American Chemical Society.

It is important to note that the H_2_ production rate under visible light illumination observed in the earlier work by Chen et al. on Black TiO_2_ was still unsatisfactory: a H_2_ evolution rate of about 0.1 mmol h^−1^ g^−1^ was determined by activation at wavelength > 400 nm, versus a 10 mmol h^−1^ g^−1^ (i.e., two order of magnitude larger) under solar illumination [236]. In order to improve the visible light-activated efficiency, a significant number of works investigated additional routes for producing more efficient black TiO_2_. A commonly employed strategy involved the presence of Pt as co-catalyst, given its very high intrinsic efficiency in H reduction, as discussed in next section. However, it is important to underline that follow-up works evidenced that hydrogenated or reduced TiO_2_ has been shown to be able to produce H_2_ even in absence of any metal cocatalysts. This point was confirmed by several works employing different samples morphologies, such as single crystals [241], powders [242] or anodic nanotubes [243,244,245]. The absence of extrinsic catalytically-active reduction sites suggest the presence of some intrinsic co-catalytic site that appeared to be closely related to the hydrogenation process. An example is shown in Figure 23, taken from reference [243]: a significant H_2_ evolution rate for anatase nanoparticles treated in high-pressure hydrogen flow was measured while other reduction processes such as high-temperature annealing in air or in Argon did not lead to significant H_2_ evolution rate under solar illumination.

**Figure 24:**
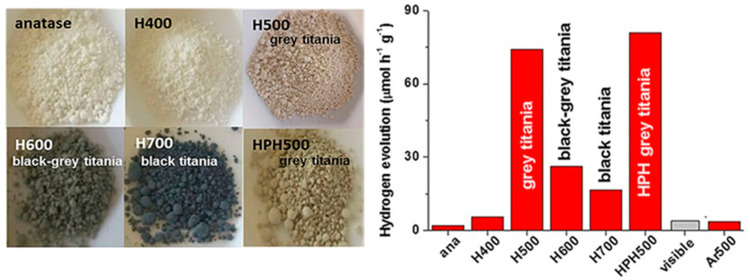
Left: Images of anatase nanopowders after different hydrogenation conditions (H: flow furnace, HPH: high pressure hydrogenation). Right: Rate of photocatalytic hydrogen evolution rate under AM 1.5 illumination for TiO_2_ nanoparticles after different hydrogenation treatments. It is to be noted here that the largest activity is obtained not for black TiO_2_ but for grey TiO_2_. Reprinted with permission from Reference [246]. Copyright 2017 Wiley-VCH Verlag GmbH & Co. KGaA, Weinheim.

In a work by Liu and coworkers [246] it was shown that no significant changes in morphology and (anatase) crystalline order occurred after the hydrogenation process. An even more striking result was the uncorrelation between the optical properties of such “grey” samples and their solar light-activated H_2_ photoproduction, as these samples exhibited negligible optical absorption and moderate values of H_2_ production rates (see Figure 24). These findings suggest that the actual responsible for the H_2_ photoevolution under solar illumination was not the entire ensemble of defects that determine the overall anatase coloration, but instead some specific type of defect, whose formation was optimized by the sample processing.

**Figure 25:**
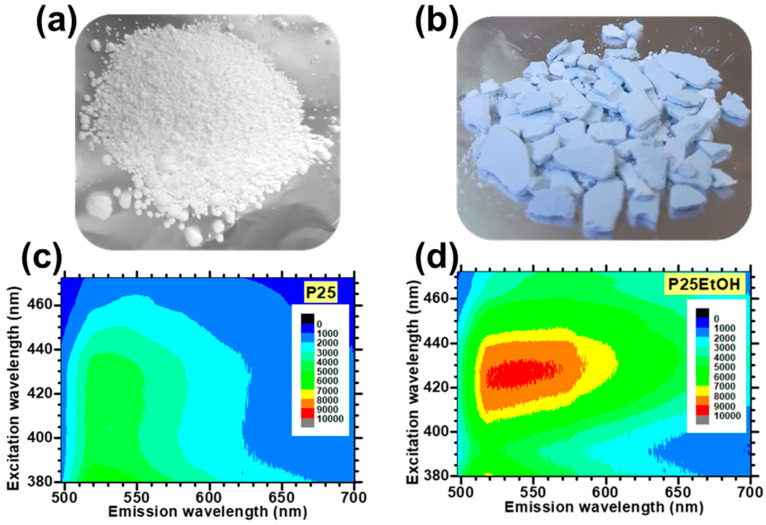
Top: images of untreated (**a**) and ethanol-treated (**b**) P25 powders. Bottom: excitation-resolved photoluminescence intensity maps (also PLE maps) for untreated (**c**) and ethanol-treated (**d**) P25 samples. The new excitation band which appears for ethanol-treated samples with a peak excitation wavelength of about 420 nm is associated to an additional radiative recombination channel that involves sub-gap excitations of valence band electrons to shallow states characterizing the reduced TiO_2_, which then relax to deeper and luminescence-active electron traps. Figure 25 (**c**,**d**) are reprinted with permission from Reference [205]_._ Copyright 2020 American Chemical Society.

Follow-up analyses based on PL measurements characterized the grey anatase H_2_-treated samples with an additional light emission in the range of 400–450 nm [247], which corresponds to sub-bandgap states positioned 0.2–0.4 eV below the conduction band edge. Interestingly, such a conclusion is not only compatible with scheme proposed in Figure 14 (shallow states) to explain the sub-bandgap excitation of PL in anatase (see processes (f) and (g)), but also is supported by a work by Lettieri and coworkers, who found a “optical signature” of reduced anatase by exploring a different strategy for the production of visible light-activated reduced TiO_2_ [205]. In this work, a simple ethanol refluxing treatment at mild temperatures of P25 nanoparticles was employed to produce “blue TiO_2_” with improved visible light photocatalytic activity. The treatment caused an increase of Ti^3+^ states and, most notably, a new sub-gap optical excitation band (absent in pristine anatase) associated to anatase PL and peaked at 430 nm, as shown in Figure 25. The data represent photoluminescence excitation (PLE) intensity maps for untreated P25 (A) and EtOH-treated samples (B and C) and the novel excitation feature is clearly visible, with a maximum efficiency for 430 nm excitation wavelength, corresponding to a photon energy of about 2.9 eV (0.3 eV below the CB edge).

In summary, different findings suggest the existence of stable and “shallow” Ti^3+^ states whose energy position is close (0.2–0.4 eV below) to the CB edge, well above that of deep traps positioned about 0.8 eV below the CB edge (see Section 4.2 and references therein). These states appear to be responsible for the cocatalyst-free H_2_ evolution by hydrogenated “colored” TiO_2_. Some indications about the actual nature of these intrinsic defects active in H_2_ production involve their location in the crystal: for example, investigations performed on anatase single-crystals correlated the H_2_ photocatalytic production of hydrogenated TiO_2_ to was associated to the presence of surface defects on high-index planes [241]. Additional procedures have been experimented to produce colored TiO_2_ with photocatalytically-active intrinsic defects. Some examples include H^+^ ion implantation [244], photodegradation of organic molecules on TiO_2_ surface [205,248,249], different treatments with reducing agents [250,251,252] and others: due to the vastness of the topic, the reader is referred to specialized reviews [22,233,238,253] for additional information.

#### 7.1.2. Facet Engineered TiO_2_

It is well established that the surface termination of a single-crystal metal oxide semiconductor affects several important electronic parameters, such as energy levels, bandgap energies and work functions. Several phenomena of facet-dependent properties in oxides of interest for photocatalysis are documented for ZnO and TiO_2_. The case of ZnO is a representative one: it crystallizes in polar (wurtzite) structures made of O^2−^ and Zn^2+^ planes perpendicular to the c-axis and hence its surface consists of a positively charged Zn-terminated (002) planes and a negatively charged O-terminated (002) planes. This facilitates to discern surface termination-dependent phenomena and study related phenomena. An example is a study by Wong et al. who detected via confocal microscopy the green PL emission (~520 nm) which is mostly correlated to surface oxygen vacancies, concluding that these defects in ZnO surfaces preferentially form along the polar (002) facets instead than on the non-polar (100) ones [254].

**Figure 26:**
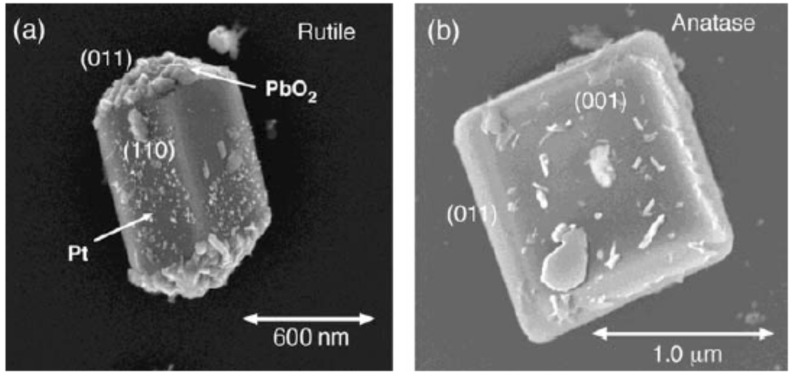
SEM images of a rutile particle (**a**) and an anatase particle (**b**) showing surface-selective PbO_2_ deposits. Reprinted with permission from Reference [255]. Copyright 2002 Royal Society of Chemistry and Centre National de la Recherche Scientifique.

The interest in facet engineering in TiO_2_ nanocrystals arises from the possibility to improve the photocatalytic efficiency by using the different ability of non-equivalent surface planes to trap electrons instead than holes and thus achieve an effective charge carrier separation without the need to employ heterojunction effects and cocatalysts. An earlier evidence of the actual occurrence of surface-selective accumulation of electrons in single-crystal TiO_2_ has been reported by Ohno et al., who observed a selective photodeposition efficiency for Pt and PbO_2_ on specific surface-oriented anatase and rutile crystals [255], as shown in Figure 26.

Analysis of these results pointed toward a preferential transfer of electrons toward the TiO_2_ (101) facets, while instead holes are driven toward the (001) surface. In other words, they suggested that once photogenerated charge carriers of opposite sign reach the surface of an anatase crystal, its (101) surface will provide reduction sites while its (001) surface will provide oxidation sites.

**Figure 27:**
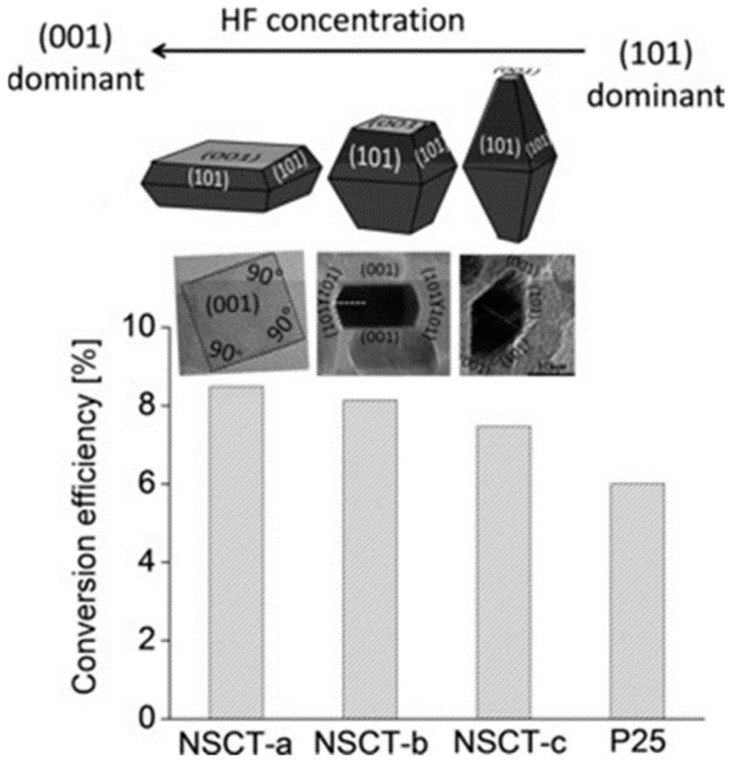
Graphical representation of the photoconversion efficiency vs. different percentage of exposed (001) facets in anatase TiO_2_ nanocrystals. Reprinted from Reference [262]. Copyright 2011 John Wiley and Sons.

A factor that typically limits the possibility to employ surfaces with high reactivities is that their relative amount tends to decrease as the crystal grows, due to the natural tendency to minimization of the surface energy. In the case of anatase TiO_2_ crystals this tendency translates in the predominance of (101) facets, which are more thermodynamical stability than the (001) ones. According to the Wulf construction [256], the relative amount of (101) is expected to be about 94% in an anatase crystal. Hence, the spatial charge carrier separation suggested by the results of Ref. [255] and the related eventual improvement of photocatalytic efficiency caused by the coexistence of both the facets is difficult to be observed in normal conditions. Therefore, the demonstration by Yang and coworkers [257] that in fluorine-terminated TiO_2_ surfaces the relative stability of the two surfaces is inverted, i.e., (001) becomes energetically favored with respect to (101) was in important breakthrough, as it provided synthetic procedures for producing nanocrystals with co-exposed (001) and (101) facets and controlled shapes (see also Figure 27).

Approximately in the same period, other procedures for growing TiO_2_ nanocrystals with controlled shapes such as spherical, rhombic, elongated rhombic, truncated rhombic and dog-bone shapes were published by Dinh et al. [258], based on modified solvothermal techniques that employ oleylamine and oleic acid as capping surfactants (for the technical details we refer the interested reader to the original work, Ref. [258]).

Since these promising results, the role played by differently-oriented surfaces in TiO_2_ [234,259,260,261,262,263,264,265,266,267,268] and in other catalysts such as Cu_2_O [269], ZnO [254], WO_3_ [270] and BiVO_4_ [271] has been theoretically and experimentally studied.

Regarding TiO_2_, several works involved the study of facet-dependent photocatalytic reactions on anatase with co-exposed (001) and (101) facets.

An interesting technical approach for those kind of studies has been reported by Tachikawa and coworkers [261], who shown that single-molecule fluorescence imaging of redox-responsive dyes can be used as spatially-resolved technique to evidence the different reactivities of (101) vs. (101) anatase facets, where the latter was the preferential site for photoinduced dye reduction [261]. This conclusion is supported by the work published in the same year by D’Arienzo et al. [259] employed photogeneration and analysis by electron spin resonance (ESR) of defects in shape-controlled anatase nanocrystals to address how the (001) and (101) facets participate to redox processes. In more detail, the exposure to UV radiation determined the formation of Ti^3+^, O^−^ and O^2−^ charge-trapping centers which are detectable by ESR. Correlating the relative amount of exposed crystal facets of nanocrystals with the relative abundance of these defects and with the sample reactivity (via phenol photodegradation). The experiments indicated that samples with larger relative amount of (001) surface area also exhibited larger concentrations of trapped holes and of photo-oxidation, while lesser photo-oxidative efficiency was associated to larger relative amounts of (101) surface areas and to Ti^3+^ centers. The authors therefore concluded that the (001) and (101) surfaces essentially act as oxidation and reduction sites, respectively [259].

**Figure 28:**
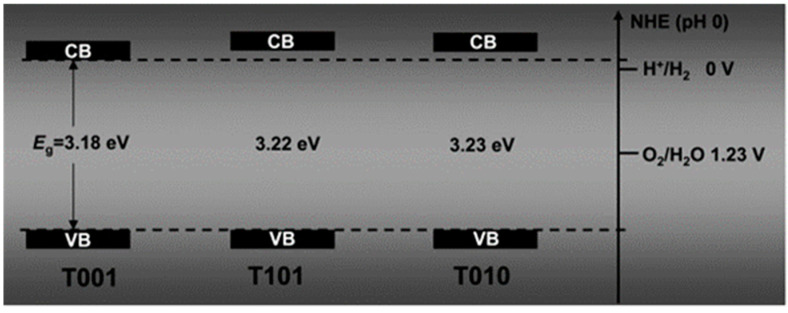
Bandgap energies extracted by optical absorption data from nanosized anatase single crystals with predominant (100)}, (001), and (010) exposed facets and compared with the standard semireactions potential for water oxidation and hydrogen reduction. Reprinted with permission from Reference [260]. Copyright 2011 John Wiley and Sons.

While the latter examples basically agree each other, other works pointed out that the surface characteristics are not intrinsically sufficient to determine the photo-oxidation reactivity. For example, Pan and coworkers [260] analyzed (001), (101) and (010)-terminated nanocrystals and argued that it is the combination of surface atomic structure (namely: the density of under-coordinated Ti_5c_ surface atoms) and of band structure (namely: the energy position of conduction band edge) that defines the actual photo-oxidation efficiency of the anatase nanocrystal. In more detail, the authors stated that the (001) and (010)-terminated crystals are favored in terms of photo-oxidative efficiency by a high density of Ti_5c_ sites, while the (101) and (010)-terminated crystals express exhibit stronger photo-reductive efficiency due to a higher energy of the electrons at the minimum of the conduction band, represented schematically in Figure 28. Since an efficient electron consumption can at the same time hamper the charge carrier recombination and thus promote photooxidation reactions initiated by the holes in the valence band, the authors concluded that (010) are expected to exhibit the highest reactivity.

**Figure 29:**
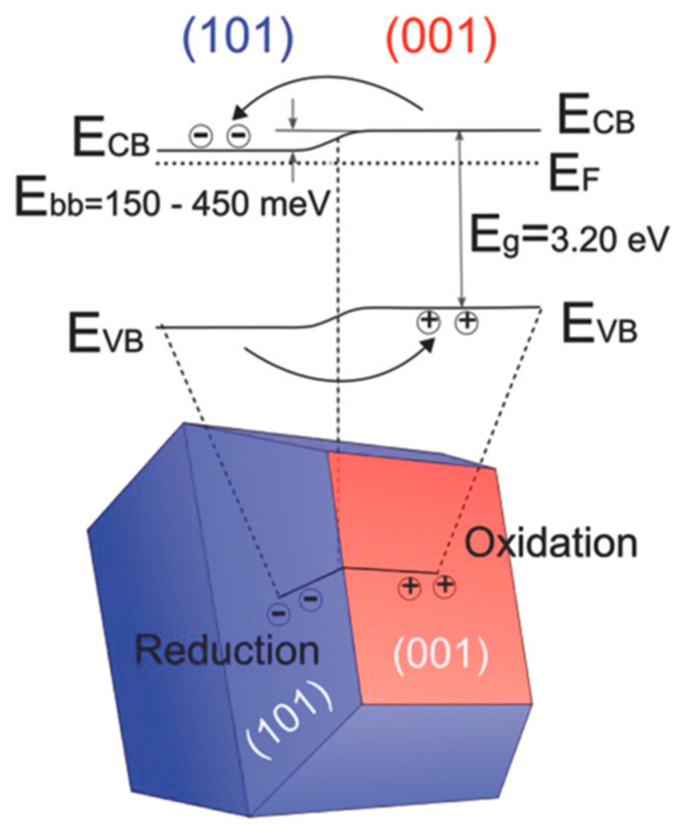
Interfacial band bending and spatial separation of charge carriers on anatase crystallites with co-exposed (001) and (101) facets. The band bending at the interface between the facets is based on the experimentally determined Fermi level difference between the facets and induces a transfer of photogenerated electrons from the (001) surface to the (101) surface (and vice-versa for the photogenerated holes). Reprinted with permission from Reference [267]. Copyright 2018 John Wiley and Sons.

Insights in the origin of enhanced photocatalytic activity of facet-engineered anatase were experimentally evidenced by Kashiwaya et al. who prepared well-defined (001)-terminated and (101)-terminated anatase by means of different procedures and determined their Fermi level position by ultraviolet photoelectron spectroscopy [267]. The experimental results indicated that, regardless the surface preparation (sputtered vs. annealed) and the sample state (stoichiometric vs. oxidized), the Fermi level of (001) facets always had energy values lower (by amounts of 150–450 meV) than that of (101) facets. This Fermi level offset determines a band bending at the boundary between the two facets, acting as an effective “driving field” for spatial separation of the charge carriers. A schematic representation of the processes that shall occur in a faceted anatase crystal are shown in Figure 29.

Application of facet-engineered TiO_2_ for photocatalytic-based applications has been demonstrated in several works. For example, we mention here the improved H_2_ generation yield via methanol photosteam reforming published by D’Arienzo and coworkers [266] who also evidenced the reactivity of the (010) facets, a topic that was also highlighted in the Ref. [260] discussed previously. Other examples involve the enhanced efficiency of dye-sensitized solar cells (DSSCs) using photoanodes made of TiO_2_ nanocrystals with high percentage of exposed (001) facets [262] and the improvement of CO_2_ reduction [265] by anatase TiO_2_ with co-exposed (101) and (001) surfaces.

Other references and application examples can be found in reviews specialized on the topic [234,263,264,268].

### 7.2. TiO_2_-Based Heterojunction Photocatalysts

The coupling of TiO_2_ with a different and suitable material can be a way to tackle either the problem of visible (sunlight) activation and/or that of charge separation. In fact, a suitable “partner” material—also referred to as cocatalyst—may form a heterojunction with TiO_2_, inducing a suitable built-in electrostatic fields and internal driving forces that facilitate the spatial separate the opposite charge carriers.

In other cases, the partner material could undertake the role of absorbing the photons in the visible range and of injecting the resulting photogenerated charge carriers into TiO_2_. In regard to this approach the topic of dye-sensitized TiO_2_ materials is worth mentioning (see Section 7.2.4).

For a useful classification, we can distinguish between the cases in which the cocatalyst is a conductor (i.e., a metal) or a semiconductor. In the latter case, we can further make a distinction for metal oxide semiconductors and other classes of semiconductors. A separate class consists of carbonaceous and graphene-related cocatalysts, which may exhibit different degrees of semiconductor characteristics. This latter class includes for example graphene and reduced graphene oxide, carbon quantum dots and graphitic carbon nitride (g-C_3_N_4_). As the subject of graphene-TiO_2_ systems is too vast to be discussed here, we will briefly introduce only the topic of TiO_2_/g-C_3_N_4_ composites as it has been receiving a lot of attention in the latest years.

Metallic (or bimetallic) cocatalysts are used in conjunction with TiO_2_ as they typically contribute to spatial separation of photogenerated charge carriers and can favor the activation by visible light through the exploitation of plasmonic resonances, as it will be discussed in Section 7.2.1.

**Figure 30:**
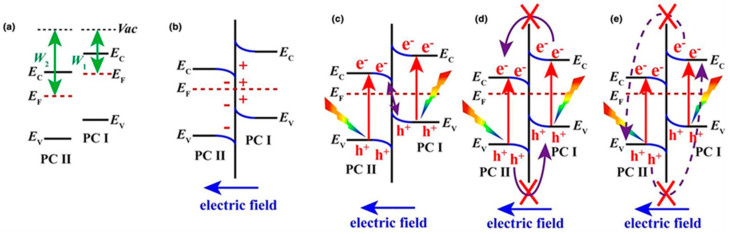
(**a**) Representation of the energy level configuration for a direct Z-scheme heterojunction. The photocatalyst on the left (PC II) was lower conduction band edge energy and higher work function (W_2_) than the photocatalyst PC I. This leads to the built-in electric field and band bending represented in (**b**). Under photon absorption the photogenerated electrons of PC II and holes of PC I accumulate at the interfacial region, increasing the probability of mutual recombination and thus the effective charge separation (**c**), while the unfavorable charge transfer processes or recombination indicated in (**d**) and (**e**) respectively are hampered by the relative position of the band edges and by the built-in electric field. As discussed in the text, this configuration is ideal to increase the redox efficiency of the composite system when the PC II is a strongly oxidizing photocatalyst such as TiO_2_. Adapted with permission from Ref. [235]. Copyright 2018 Elsevier.

Regarding semiconductor-based photo-reactive cocatalysts, the most desired and advantageous configuration in which they can form a heterojunction with TiO_2_ is the one referred to as “direct Z-scheme configuration”, represented in Figure 30. In more detail, the direct Z-scheme is achievable if the semiconductor with higher value of conduction band edge (or more precisely, with larger electron affinity) has also the lower value of the work function, as indicated in Figure (where the work functions are indicated by W_2_ > W_1_). This might for example happen when the Fermi levels are close to the middle of the bandgap (as in undoped semiconductors). In this case, a built-in electric field will be formed at the interfacial region, leading to a band bending as represented in Figure 30, so that excess electrons generated in the semiconductor with lower work function (PC I in Figure, where “PC” stands for photocatalyst) will tend to remain in it, as a potential barrier at the interface prevents the transfer toward the other semiconductor. The same conclusion can of course be derived for the photogenerated holes in the semiconductor with larger work function (PC II in Figure). Therefore, the system will tend to remain for longer times with non-recombined holes in PC II and electrons in PC I. This is an ideal situation for cases in which PC II is TiO_2_ and PC I is a visible light-absorbing photocatalytic semiconductor.

In fact, in such cases holes will remain longer in TiO_2_ which has a large oxidative efficiency (and larger than holes in PC I), while electrons in the partner material (PC I) have a larger reductive efficiency than electrons in PC II (In terms of redox potential, this is because the electrostatic potential is more positive than for holes in PC II than for holes in PC I, while it is more negative for electrons in PC I than for electrons in PC II). This situation presents a definite advantage for photocatalytic processes in order to overcome the problems related to the scarce charge separation and short charge-carrier lifetime in intrinsic TiO_2_.

#### 7.2.1. TiO_2_/Metal Heterostructures

Metals are probably the most commonly employed materials used to improve the photocatalytic properties of TiO_2_. Both noble metals (such as Pt, Au, Pd, Ag) and non-noble metals (such as Cu and Ni) are commonly used as cocatalysts. Their role can be stated by considering three basic functions, namely: (i) Favoring the charge separation; (ii): Improving the optical absorption and local electromagnetic in the visible range via excitation of plasmonic resonances, and (iii) Providing additional catalytic active sites (i.e., the metal itself acts as catalyst) on the surface of the TiO_2_ nanoparticle.

**Figure 31:**
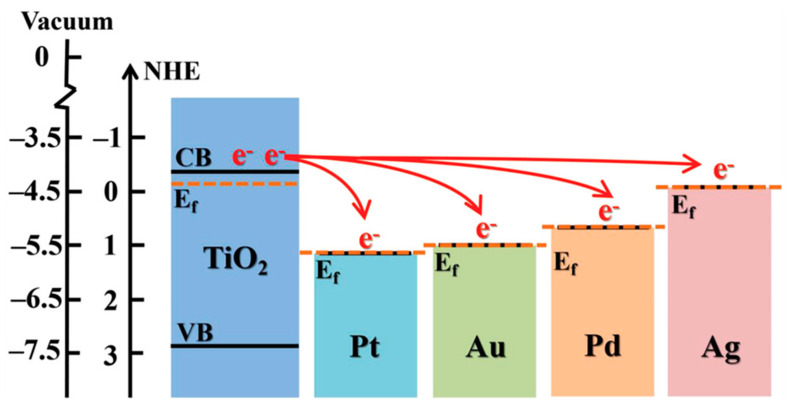
Potential energy values (referenced to the vacuum level) and electrical potential values (referenced to NHE) for the conduction band edge of TiO_2_ compared with the Fermi levels of Pt, Au, Pd, and Ag. The latter values refer to cases where the metals are isolated (that is, in absence of electrical contacts with other materials and in absence of illumination). Reprinted with permission from Reference [21]. Copyright 2019 WILEY-VCH Verlag GmbH & Co. KGaA, Weinheim.

The charge separation is caused by the built-in electric field that occurs close to a metal/semiconductor Schottky junction, that in turn is caused by the transfer of free charge carriers between two materials with different work functions. If the physical contact between a metal and a semiconductor is close enough to permit the quantum coupling of the charges of the two materials (i.e., in first approximation when spatial overlap of the orbitals of the charge carrier is present), electrons will transfer from the semiconductor to the metal when the Fermi level is lower in energy than the semiconductor one or—to state it in different but equivalent words—when the work function of the semiconductor is lesser than that of the metal. Metals typically have larger work function (W_f_) than metal oxide semiconductors, as their values typically span in the 4–5 eV range and with notable values of about W_f_ ≈ 5.6 eV for Pt and W_f_ ≈ 5.1 eV for Au, Pd and Ni [272]. These values indicate that electron transfer from TiO_2_ to the metal is likely to occur, as they indicate Fermi levels of the metal which is well below in potential energy (and more positive in terms of electric potential vs. NHE) of the conduction band edge of TiO_2_. See Figure 31 for reference.

In other words, as the photocatalyst is activated by optical absorption the transition region close to the heterojunction will be depleted of electrons, due to the effect of the built-in electric field and the electron-hole recombination will be slowed down. The effect is more pronounced for higher Schottky barriers, which based on the data shown in Figure occur at contact of TiO_2_ with Pt and Au.

The metal itself also typically contributes to a catalytic process. For example, Pt is very effective for hydrogen evolution (i.e., proton reduction) due to the fact that the Gibbs energy change for atomic hydrogen adsorption on Pt is very low, so that the process in which Pt binds to H+ and releases H_2_ is thermodynamically easier than for example for the case of Au [273]. However, Au has a distinct advantage in that it supports localized plasmonic resonance in the visible range [274,275]. An example showing a direct evidence of plasmonic-related improvement of photocatalytic efficiency in TiO_2_/Au systems was shown in Reference [276], where the photocatalytic production rate for H_2_ under illumination of TiO_2_ nanofibers co-decorated by Pt and Au was measured for optical excitation at 550 nm (plasmon resonance for the Au particles) and 420 nm. The experiments evidenced a wavelength-dependent enhancement for H2 generation correlated to the plasmonic absorption of Au.

The plasmonic properties of Au allow to employ it in combination with a different metal, in order to take advantage of both Schottky junction-related charge separation and plasmonic coupling with optical radiation. Therefore, Au–Pt or similar (Au–M) bimetallic cocatalyst (M can typically be also Pd, Ag, Ru, Rh) are often employed to improve the photocatalytic efficiencies of TiO_2_ [277,278,279,280].

#### 7.2.2. TiO_2_/Metal Oxide Semiconductors 

Several metal oxide semiconductors can be used as cocatalysts having the function of trapping either electrons or holes photogenerated in TiO_2_. Examples include oxides of copper such as CuO and Cu_2_O, NiO, RuO_2_, IrO_2_ and oxides of cobalt (CoO_x_).

**Figure 32:**
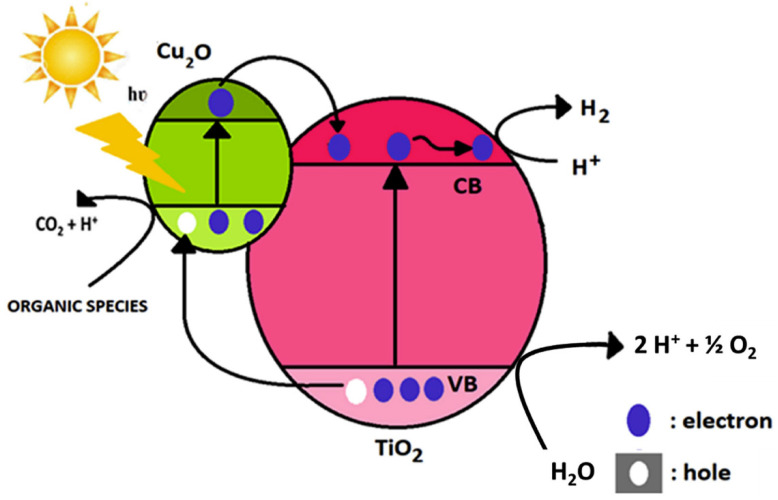
Scheme of the hydrogen evolution process in a Cu_2_O/TiO_2_ heterojunction photocatalyst under solar illumination. It is assumed that the Fermi level equilibrium has already been achieved, to that the built-in field drives electrons from Cu_2_O to TiO_2_ and vice-versa. The scheme includes possible photogeneration in both TiO_2_ and Cu_2_O, where the latter implies the possibility of H_2_ generation activated by visible light thanks to the electron transfer from Cu_2_O to TiO_2_. Reprinted with permission from Reference [287]. Copyright 2020 Hydrogen Energy Publications LLC. Published by Elsevier Ltd.

Cuprous oxide (Cu_2_O) is often employed as reduction cocatalyst: it is a p-type semiconductor with a narrow bandgap of 2.2 eV. As TiO_2_ is a n-type semiconductor, a TiO_2_/Cu_2_O junction typically results in a p-n heterojunction and, at equilibrium, in a built-in electrostatic field that drives excess (photogenerated) holes electrons of TiO_2_ toward Cu_2_O (and vice-versa for electrons photogenerated in Cu_2_O). As a result, Cu_2_O can be effectively used as reduction cocatalyst in hydrogen for photocatalytic hydrogen evolution [281,282,283,284,285]. A schematic representation of the H_2_ evolution reactions over a TiO_2_/Cu_2_O composite is reported in Figure 32. The scheme shows the oxidation of water (or of an organic species) operated by holes in Cu_2_O, with proton reduction by photogenerated electrons in the conduction band. For simplicity, the scheme assumes here the generation of charge carriers in both the materials and thus shows the both the possible charge transfer pathways. Noticeably, the narrow optical gap of Cu_2_O permits the activation of proton reduction under visible light illumination: the energy offset in the conduction bands (i.e., −1.5 Volts and −0.5 Volts (vs. NHE) for Cu_2_O and TiO_2_ respectively [286] implies that the electron are transferred to TiO_2_, where the proton reduction can occur as shown in Figure, while photogenerated holes remain in Cu_2_O where they participate to the oxidation of water or of an organic species that acts as suitable sacrificial agent.

Cuprous oxide is often used in conjunction with additional functional elements, such as noble metals (e.g., Pt [288,289], Au [290] or metal copper [291,292,293,294]) to prepare ternary hybrid photocatalysts. A review that includes several references and experimental results on the use of TiO_2_/Cu_2_O composites for H_2_ production through photoreforming processes has been recently published by Marotta and coworkers [287].

**Figure 33:**
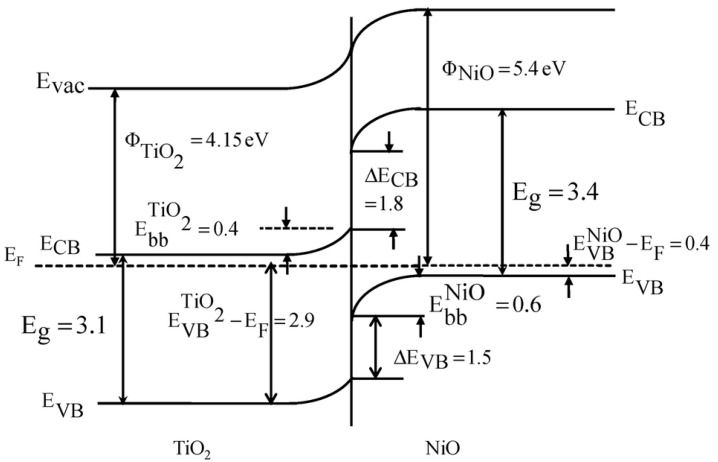
Band alignment at the TiO_2_/NiO heterojunction. Reprinted with permission from Reference [295]. Copyright 2017 Royal Society of Chemistry.

Nickel oxide (NiO) is also a p-type semiconductor which can be employed to obtain p-n heterojunctions with TiO_2_. An accurate characterization of the energy levels alignment of NiO/TiO_2_ composites has been reported by Uddin and coworkers [295], who combined XPS, UPS and absorption spectroscopy to sketch the electronic configuration of the p-n heterojunction as shown in Figure 33. The resulting heterojunction photocatalysts typically exhibit an improved efficiency (compared to commercial anatase or P25 nanoparticles) in oxidative photodegradation of pollutants [296] and hydrogen evolution [295,297].

For additional information on other metal oxide cocatalysts−such as RuO_2_, Co_3_O_4_ and IrO_2_, the reader may consult the review by Meng and coauthors [21] and references therein.

#### 7.2.3. TiO_2_/g-C_3_N_4_ Heterostructures

In recent years, graphitic carbon nitride (g-C_3_N_4_) has become one of the newest and mostly investigated low-gap photocatalytic materials, both as a stand-alone photosensitive 2D material and as a functional “partner” for TiO_2_/g-C_3_N_4_ heterostructures for hydrogen production.

**Figure 34:**
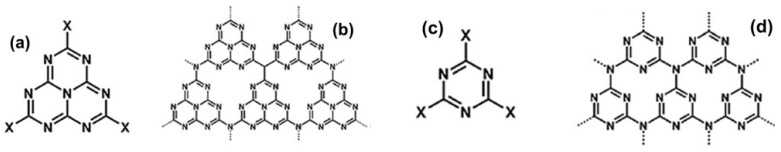
(**a**) s-heptazine (or tri-s-triazine), (**b**) s-heptazine-derived g-C_3_N_4_, (**c**) s-triazine, (**d**) s-heptazine-derived g-C_3_N_4_. Adapted from Reference [300] with permission. Copyright 2015 WILEY-VCH Verlag GmbH & Co. KGaA, Weinheim.

The term graphitic carbon nitride (g-C_3_N_4_) designs a class of carbon nitride compounds whose general formula is C_3_N_4_, although it is common that the stoichiometric ratio is slightly modified due to some amount of hydrogen. The class includes seven phases of C_3_N_4_ [298], two of which are more interesting for photocatalytic applications. These are the g-h-triazine phase, obtained by polymerization of s-triazine (Figure 34a) and the g-h-heptazine phase, obtained by polymerization of tri-s-triazine (or heptazine, Figure 34b). Their energy gaps were calculated to be 2.97 and 2.88 eV, respectively, while instead the other forms of C_3_N_4_ show optical absorption only in the ultraviolet range [299]. As a consequence, the g-h-triazine and g-h-heptazine phases are those suitable for photocatalytic production of hydrogen.

The interest in g-C_3_N_4_ was boosted by the observation of H_2_ production via water splitting under visible light illumination by Wang et al. in 2009 [301]. The interest in the material has increased since then, prompted by the potential perspective of conjugated polymers shifting the dominance among photocatalysts from inorganic semiconductors to more abundant and easily processable polymeric ones [302,303]. The material in fact shows several advantages: it is fairly inexpensive, highly available, chemically inert in several solvents, thermally stable (up to 600 °C in air). Furthermore, as mentioned previously, its optical gap allows the optical absorption of visible light. Finally, bulk powders can be used to prepare few-layers or even single-layer 2D material.

Exploitation of heterojunction effects of g-C_3_N_4_ with TiO_2_ clearly represents a route to be explored in order to improve the performances of both the materials. While in fact the g-C_3_N_4_ is capable to add the visible-light activation functionality to TiO_2_, this latter is more effective as oxidating agent due to the relative position of its valence band with respect to the one of g-C_3_N_4_.

**Figure 35:**
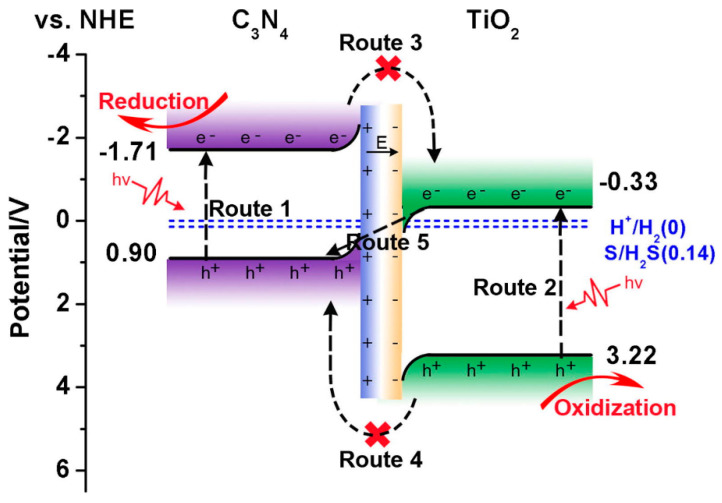
Electronic configuration of a TiO_2_/g-C_3_N_4_ heterojunction with direct Z-scheme configuration. The various processes (Route 1 to Route 5) and the favorable separation of photogenerated holes in TiO_2_ and electrons in g-C_3_N_4_ is discussed in the text. Reprinted from Reference [304] with permission. Copyright 2019 Elsevier.

Several theoretical and experimental works point out that the electronic coupling of g-C_3_N_4_ with TiO_2_ supports the occurrence of a direct Z-scheme band alignment, as described in Figure 30. An example is shown from the work by Wei and coworkers [304], whose computation of work function of the separated materials and of the band alignment of the g-C_3_N_4_/TiO_2_ led the authors to conclude that the equalization of the Fermi levels of the two materials sets up a built-in electric field as in Figure 35 and representative of a direct Z-scheme configuration. In fact, the configuration prevents photogenerated electrons in g-C_3_N_4_ and holes in TiO_2_ to cross the interface, as shown in Figure for the processes labelled as “Route 3” and “Route 4” which are electrostatically inhibited. On another hand, the recombination of photogenerated TiO_2_ electrons and C_3_N_4_ holes is possible and electrostatically favored near the interface (Route 5).

Nowadays, significant efforts are put in the study of TiO_2_/g-C_3_N_4_ heterostructures for applications such as H_2_ photogeneration, CO_2_ reduction and pollutants photodegradation. Perspectives are generally considered promising, as proved by the large amount of works published in the topic in the last years, even though some aspects need to be improved such as adsorption affinity for pollutants and long-term reproducibility of photocatalyzed photodegradations [303]. For additional information the reader is referred to some specialized review on the topic [302,303,304,305,306] and references therein.

#### 7.2.4. Dye-Sensitized TiO_2_

Coupling TiO_2_ with organic dyes capable to absorb light in the visible range and inject the photogenerated electrons to TiO_2_ represents a possible way to achieve a sunlight-activated photocatalyst. A coupled TiO_2_/dye system in which such two-step sequence (i.e., photon absorption and charge carrier transfer) occurs is known as “dye-sensitized” TiO_2_. Dye sensitization in TiO_2_ is a well-known topic, being it the key ingredient for the realization of dye-sensitized solar cells (DSSC), a well-known and widely studied class of thin-film photovoltaic cells that are receiving worldwide attention since more than two decades thanks to their simple preparation, low toxicity and affordable production costs. Even is the topic of DSSCs is beyond the scope of this review, for the sake of our discussion it is worth mentioning that electrons in DSSCs are generated by optical absorption in the dye molecule and transferred to a TiO_2_ photo-anode, while holes are transferred to a reduced form (typically an iodide ion I^−^) of redox mediator which is then reduced back by gaining electrons at the cathode and so closing the electric circuit [307].

The interfacial charge transfer in dye-sensitized TiO_2_ (and in other dye-sensitized photocatalysts as well) occurs as in DSSC systems and electrons are then transferred to a catalyst (typically Pt) instead of being used for electricity generation (as in a solar cells). To allow the process to continue, the oxidized dye molecule has to be reduced by an appropriate electron donor (or, equivalently, a hole scavenger).

**Figure 36:**
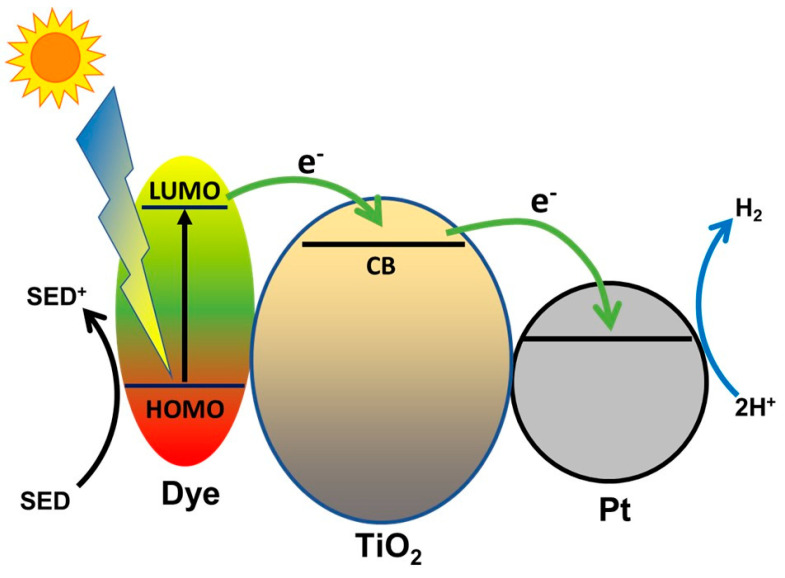
Scheme of the key charge carrier processes in H_2_ photogeneration by a dye-sensitized TiO_2_ photocatalyst. The electrocatalyst involved in the final step is here represented as platinum (Pt) in accordance with the Equation (19).

When the overall process is optimized, the dye-sensitized TiO_2_ photocatalyst can be used for solar fuel (hydrogen) generation, according to the scheme reported in Figure 36 that shows the complete sequence of elementary charge carrier processes for H_2_ production. The photoexcitation through visible light consists of the generation of electrons and holes in the dye molecules. It is worth noting the presence of a suitable “sacrificial electron donor” (indicated as SED) as reducing agent, whose role is to restore the fundamental state of the Dye molecule [308].

The sequence of elementary processes which is regarded as the one occurring more frequently involves the injection of an electron from the photoexcited dye ($D^{*}$) to the TiO_2_ as first step, implying that the excited dye becomes a dye radical cation ($D^{*+}$). Successively, the latter is reduced by the SED agent. Overall, the processes can be summarized as follows:

(17) $$Pt/TiO_{2}/D^{*}\to Pt/TiO_{2}\left(e^{-}\right)/D^{*+}$$

(18) $$Pt/TiO_{2}\left(e^{-}\right)/D^{*+}+SED\to Pt/TiO_{2}/D+SED*$$

Another scheme is possible in which the first process is now the hole capture operated by the SED agent, followed by the injection of a photoelectron from the radical anion to the TiO_2_. As mentioned, this different sequence shall occur in a minority of cases [309]. Regardless, the resulting state is the same, that is free electrons in the semiconductor CB will allow the generation of H_2_ via proton reduction:

(19) $$Pt/TiO_{2}\left(e^{-}\right)/D+H^{+}\to Pt/TiO_{2}/D+\frac{1}{2}H_{2}$$

**Figure 37:**
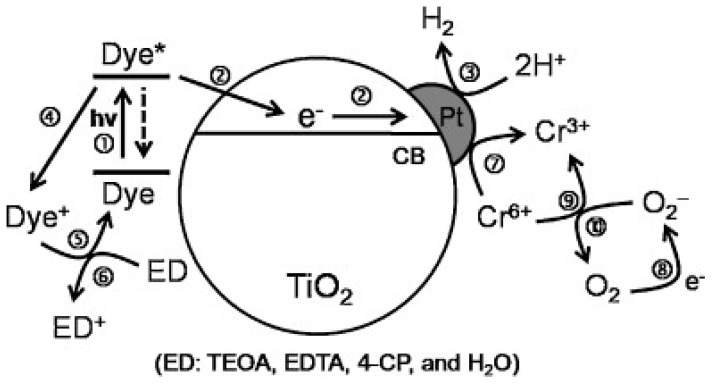
Schematic representation of photoinduced H_2_ generation and of reduction of CR(IV) pollutant through dye-sensitized TiO_2_ in water. Some particular processes to be noted are dye regeneration (processes 5 and 6) through the electron donor ED (indicated as “SED” in the previous figure), the reduction of Cr(VI) to Cr(III) by direct reduction (process 7) of by reaction with superoxide ion intermediates (process 9 and 10). Reprinted from Reference [310] with permission from Elsevier. Copyright 2012 Elsevier Science B.V.

The same class of materials can be used for remediation of environmental pollutants. For example, Choi and coworkers [310] studied the use of dye-sensitized TiO_2_ for both H_2_ generation and photodegradation through visible light of Cr(VI), as represented in Figure 37. Here, the authors evidenced as major photodegradation process the direct reduction of Cr(VI) to Cr(III) carried out by photogenerated electrons (see mechanism 7), and an indirect reaction pathway based on superoxide-mediated reduction (see mechanisms 9 and 10 in Figure). As pointed out by the authors, the photodegradation pathway for aqueous substrates involve different reactions and are more difficult to control as compared with those for H2 production that occurs predominantly in the absence of reactive oxygen species. As a consequence, the relationship between the concentrations of species and the rate of reaction for degradation of pollutants and for H_2_ generation are different, in the general cases.

It is worth mentioning that study of dyes used to sensitize photocatalysts does not overlap that of dyes for DSSCs. Instead, the two study fields are developing independently, as a direct consequence of the different conditions in which the two system operate. To this regard, we can mention that dye-sensitized TiO_2_ has to be optimized for operating in water and that the dye must allow the regeneration of the electron donor agents, which are typically different from the redox couples (e.g., I^−^/I^3−^) used in DSSC.

At present, the solar-to-hydrogen efficiency of dye-sensitized TiO_2_ is lesser than that obtainable by photoelectrochemical cells. However, the dye-sensitization approach for TiO_2_ leads to comparatively simpler, less expensive and more scalable systems [311]. Therefore, it is significant to pinpoint any room for possible improvements, in particular for what regards efficiency and cost issues. In regard to the efficiency considerations, the molecular design of the dye sensitizer is a central element. It involves the chemical structure of the groups that anchor the dye to the TiO_2_ [312], the dye hydrophobicity and hydrophilicity [313] and of course the relative energy level position with respect to the CB of the TiO_2_ (or, in more general extent, of other photocatalysts). Further information on the foreseeable progress on those aspects can be found on recent reviews [308,314,315].

## 8. Conclusions

As a conclusion of this work, we intend to underline the importance of reviewing (and, when possible or needed, updating) the basic knowledge on fundamental processes that characterize a functional photocatalyst, even if it is probable that future applications will employ it in significantly modified versions, such as highly reduced (or doped) forms or as partner material in composite systems and photocatalysts.

The literature on TiO_2_ and TiO_2_-based systems is abundant, as argued in Section 1, Section 2 and Section 3 where we also discussed some historical facts about the study of intrinsic TiO_2_ photophysical and photo-oxidating properties. As a consequence we believe that a review might be useful for scholars (in particular for those who are approaching the field) if it aims at summarizing specific and basic topics, as we did here for the photogenerated charge carrier processes in TiO_2._ The fate and the time dynamics of photogenerated electrons and holes define in a major way the applicative potential of a photocatalyst. We discussed these topics alongside the review, and in particular in Section 4 and Section 6. The optical properties are also important: they are not only responsible for the activation of the photocatalyst (absorption coefficient), but they can also be used as modifiable parameters for photocatalytic or chemical sensing applications. This point has been discussed in Section 5, underlining the possibility of using—to these aims—special features of light propagation in engineered structures (e.g., “metasurfaces” and photonic crystals) and photoluminescence of mixed-phase TiO_2_.

Mechanisms and characteristic lifetimes of TiO_2_ photocatalysis have been summarized in Section 6, discussing them in order to highlight the limitations of intrinsic TiO_2_ and introduce some of the future trends that will likely involve TiO_2_ as key element in composite catalysts. Several methods are under scrutiny to develop improved photocatalysts that might facilitate some significant advancement in solar fuel technologies. Some of these, discussed in this review (Section 7), include defect tailoring/engineering and design of nanocomposite heterojunctions. Considering this last point, it is very likely for example that research on some specific systems, such as TiO_2_/g-C_3_N_4_ will grow in the next future. Hopefully, these studies on clean energy conversion technologies will evolve in mature key-enabling technologies for the era of ecological transition.

## Figures and Tables

**Table 1:** Bulk properties of rutile and anatase TiO_2_.

Atomic Radius (nm)			Density (g/cm^3^)		
O:	0.066 (covalent)		Rutile	4.24	
Ti:	0.146 (metallic)		Anatase	3.83	
Ionic radius (nm)			Brookite	4.17	
O (−2)	0.14 nm				
Ti (+4)	0.064 nm				
Crystal structure			Lattice constants (nm)		
	System	Space group	a	b	c
Rutile	Tetragonal	P42/mnm (group #136)	0.4584	-	0.2953
Anatase	Tetragonal	I41/amd (group #141)	0.3733	-	0.937
Brookite	Orthorhombic	Pbca (group #61)	0.5436	0.9166	0.5135
Energy bandgap (eV)		Electron mobility m (cm^2^/Vs)	Dielectric constant (at room T)		
Rutile	3.0 (indirect)	Rutile: ~1 (Reference [75])		Frequency (Hz)	Value
Anatase	3.2 (indirect)	Anatase: ~10 (Reference [75])	Rutile, $⊥\hat{c}$	108	86
				104	160
			Rutile, $∥\hat{c}$	108	170
				107	100

## Data Availability

No new data were created or analyzed in this study.
